# Morphoregulatory functions of the RNA-binding motif protein 3 in cell spreading, polarity and migration

**DOI:** 10.1038/s41598-018-25668-2

**Published:** 2018-05-09

**Authors:** J. Pilotte, W. Kiosses, S. W. Chan, H. P. Makarenkova, E. Dupont-Versteegden, P. W. Vanderklish

**Affiliations:** 1 Department of Molecular Medicine, The Scripps Research Institute, La Jolla, CA 92037 United States; 2 Department of Rehabilitation Sciences, University of Kentucky, Lexington, KY 40536 United States

## Abstract

RNA-binding proteins are emerging as key regulators of transitions in cell morphology. The RNA-binding motif protein 3 (RBM3) is a cold-inducible RNA-binding protein with broadly relevant roles in cellular protection, and putative functions in cancer and development. Several findings suggest that RBM3 has morphoregulatory functions germane to its roles in these contexts. For example, RBM3 helps maintain the morphological integrity of cell protrusions during cell stress and disease. Moreover, it is highly expressed in migrating neurons of the developing brain and in cancer invadopodia, suggesting roles in migration. We here show that RBM3 regulates cell polarity, spreading and migration. RBM3 was present in spreading initiation centers, filopodia and blebs that formed during cell spreading in cell lines and primary myoblasts. Reducing RBM3 triggered exaggerated spreading, increased RhoA expression, and a loss of polarity that was rescued by Rho kinase inhibition and overexpression of CRMP2. High RBM3 expression enhanced the motility of cells migrating by a mesenchymal mode involving extension of long protrusions, whereas RBM3 knockdown slowed migration, greatly reducing the ability of cells to extend protrusions and impairing multiple processes that require directional migration. These data establish novel functions of RBM3 of potential significance to tissue repair, metastasis and development.

## Introduction

The RNA-binding motif protein 3 (RBM3), a member of small family of cold-inducible RNA-binding proteins^1–4^, regulates several aspects of mRNA metabolism and has pleiotropic functions in cell stress, development, and oncogenesis. On a molecular level, RBM3 promotes global protein synthesis^5^, the stability of mRNAs bearing AU-rich elements^6,7^, and the biogenesis of many microRNAs at the Dicer step^8,9^, functions that together suggest RBM3 exerts a broad and differential regulatory influence on the proteome. On a cellular level, early studies indicated that RBM3 plays a critical role in adaptive responses to hypothermia, where it may act as a mRNA chaperone that preserves translation capacity until the return of euthermic conditions^2,10–13^. However, it has become clear that RBM3 is induced by a wide variety of other physiological stresses (*e.g*. hypoxia, endoplasmic reticulum (ER) stress, excitotoxins, radiation, wasting, and some disease states)^14–18^. In these and other contexts, RBM3 has a strong cell protective influence. In muscle cells, induction of RBM3 opposes necrosis and apoptosis, and preserves cell morphology, in response to reactive oxygen species and atrophic conditions^19,20^. In neural cells, RBM3 protects against cell death induced by ER stress^5,18^, hypoxia/ischemia^15,21^, and varied other metabolic and disease related stresses^14,17,22–25^. Moreover, RBM3 induction has been causally linked to the neuroprotective effects of therapeutic hypothermia^22,26^. Such data point to a general role of RBM3 in cell protection. Consistent with this idea, RBM3 is the only transcript upregulated in all tissues during torpor^27^, a state involving extended hypothermia in which many tissues (including muscle and brain) are largely spared from the pathological effects of severe metabolic stress. In addition to its regulation by physiological stresses, RBM3 expression exhibits developmental and regional variation in brain^28^, and has been linked to oncogenesis as a putative proto-oncogene^6^ and predictor of the clinical prognosis of many cancers^29^.

Several observations suggest that RBM3 has morphoregulatory functions relevant to its role in cell protection and putative involvement in development and oncogenesis. RBM3 (P98179, RNPL) was among sets of RNA-binding proteins (RNA-BPs) detected in invasive pseudopodia of mesenchymal breast cancer cells^30^, and in subcellular specializations termed spreading initiation centers (SICs)^31^ that form during the initial stage of cell spreading in fibroblasts and many mesenchymal cell lines^32^. In general, several classes of RNA-BPs have been identified in SICs^31–33^ and other cell protrusions, such as podosomes^34^, pseudopodia^30,35,36^, filopodia^37^, growth cones and dendritic spines^38–40^. Their functions at these loci can be multifold, including the regulation of mRNA localization and translation events that support cell adhesion, spreading, migration, and the guidance of extensions^31–33,41^, and protein-protein interactions that regulate cytoskeletal dynamics^42^. In light of such data, the presence of RBM3 in SICs and invasive filopodia suggests that it has morphoregulatory functions in cell shape and migration, which may be important to its stress response functions and putative involvement in development and cancer. This possibility finds support in data on RBM3 expression and function in brain. In prior work, we observed that RBM3 is upregulated in brain during the early postnatal period^8^ – coinciding with the peak of synaptogenesis^43^ – and remains high in zones of proliferating and migrating cells in the adult brain; moreover, we found that RBM3 is localized to dendritic spines in mature neurons^5^. Recently, it was demonstrated that RBM3 induction confers synaptic protection in models of prion and Alzheimer’s-related neurodegeneration, whereas knockdown of RBM3 exacerbates synapse loss and deficiencies in synapse regeneration in these models^44,45^. This synapse-sparing effect of RBM3, which may be mechanistically related to synaptic regenerative capacity seen during torpor and cold shock^46,47^, supports the idea that RBM3 regulates the formation and plasticity of cell protrusions. Taken together, such data suggest RBM3 has fundamental roles in regulating the morphology and migration of cells, but this has not been fully elucidated.

Here we present evidence that RBM3 is a regulator of cell spreading, polarity and migration. We observed that RBM3 is present in multiple types of cell protrusions that form in a variety of cell lines and primary myoblasts, and that perturbation of RBM3 expression results in dramatic changes in cell polarity and spreading involving RhoA-Rho associated protein kinase (ROCK) signaling and the collapsin response mediator protein 2 (CRMP2). Consistent with the idea that these effects relate to underlying mechanisms that also regulate cell motility, we found that RBM3 promotes directional cell migration in multiple contexts via a mesenchymal-like mode involving the extension of long protrusions, whereas low RBM3 expression impairs migration and the ability of cells to extend protrusions, changing the motility of cells to a mode that in many ways resembles amoeboid migration. Taken together, the results described herein establish novel roles of RBM3 in the regulation of cell morphology, motility, and underlying RhoA pathway signaling. These effects are discussed in terms of their potential relevance to cell protection and the processes of tissue repair, development, and metastatic processes involving migration mode flexibility.

## Results

### RBM3 is present in SICs formed in different cell types and on several plating substrata

RBM3 was detected in a proteomic screen of proteins co-precipitating with vinculin and talin from MRC-5 lung fibroblasts during the early, SIC stage of cell spreading^31^. SICs are spherical outpouchings of the plasma membrane in which a ring of F-actin circumscribes a cytoplasmic interior containing some focal adhesion proteins, translation machinery, ribosomal and poly A RNA, and RNA-BPs^31–33^. They are “hot spots” of mRNA translation^32^, which is thought to promote the formation of mature focal adhesions^31,32^. Insofar as our prior work showed that RBM3 stimulates translation^5^, the detection of RBM3 in SICs raised the possibility that it is a regulator of cell spreading. However, proteomic detection of RBM3 in SICs was not validated in MRC-5 or other cells.

**Figure 1:**
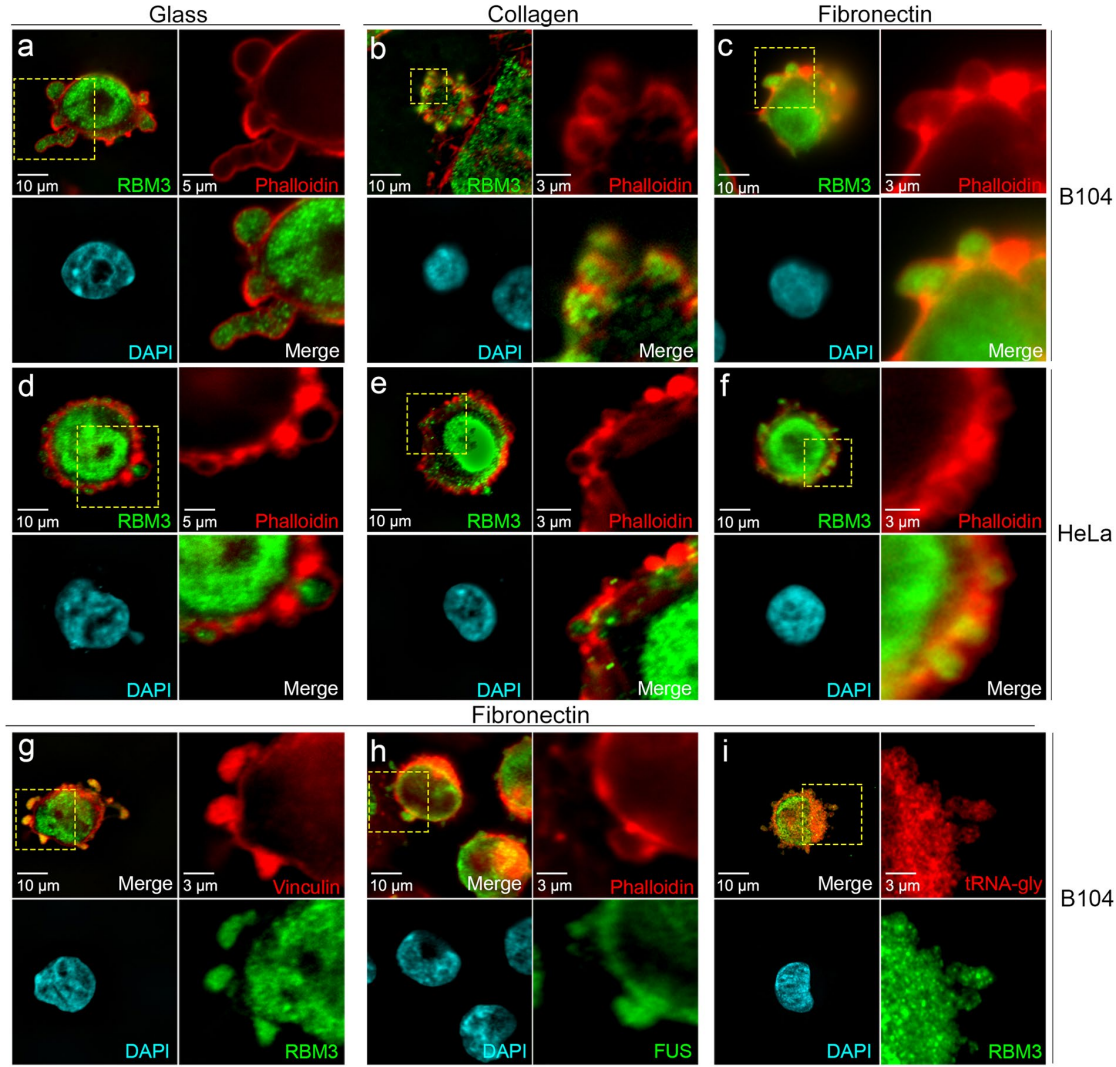
Localization of RBM3 to SICs in different cell types and plating conditions. Images of RBM3 (green), F-actin (red, phalloidin), and DAPI-stained nuclei (blue) in B104 cells (**a–c**) and HeLa cells (**d–f**) 30 minutes after plating onto glass, collagen, and fibronectin. For each cell type and substrate in panels a through f, upper right sub-panels show close-ups of F-actin distribution in regions (hatched yellow rectangles) at cell margins that contain SICs; sub-panels in lower right show the overlay of RBM3 with F-actin. (**g–i**) Images of B104 cells grown on fibronectin that were labeled for RBM3 and other SIC components by immunofluorescence, and for tRNA by FISH: cells were double labeled for (**g**) RBM3 (green) and the SIC component vinculin (red); (**h**) FUS (green) and F-actin (red); and (**i**) RBM3 (green) and tRNA-glycine (red).

To assess whether RBM3 localizes to SICs and the extent to which this is generalizable to multiple cell types and plating conditions, we monitored its distribution in different cell types after plating onto several distinct substrata (Fig. 1). RBM3 was present in SICs formed around the periphery of newly adherent cells early (30 minutes) after plating of B104 neuroblastoma cells (Fig. 1a–c) and HeLa epithelial adenocarcinoma cells (Fig. 1d–f) onto glass, collagen, and fibronectin. RBM3 staining (green) in SICs was circumscribed by a ring of F-actin stained with phalloidin (red). We confirmed that RBM3 co-localized with the cytoskeletal protein vinculin (Fig. 1g), a known component of SICs^31,32^, and that SICs contained the RNA-BP FUS as previously reported^31,32^ (Fig. 1h). In addition, we expanded the characterization of SICs by validating the presence of tRNA for glycine using fluorescent *in situ* hybridization (FISH, Fig. 1i). Repeating these studies in cells fixed 3hrs after plating, a time point when SICs are no longer present and cells are more spread, revealed that RBM3 was redistributed to the cytoplasm and nucleus in multiple cell types and plating conditions (Supplementary Fig. S1). Similar results were observed in primary myoblasts (see below) in which RBM3 was strongly localized to SICs formed initially after plating, then relocalized to the cytoplasm and nucleus after further morphological elaboration of cell shape. These data suggest that localization of RBM3 to SICs shortly after cell attachment, followed by redistribution to nuclear and cytoplasmic compartments, is a generalizable feature of adherent cells and may reflect a basic role of RBM3 in cell spreading.

### RBM3 regulates cell spreading and the development of polarity

**Figure 2:**
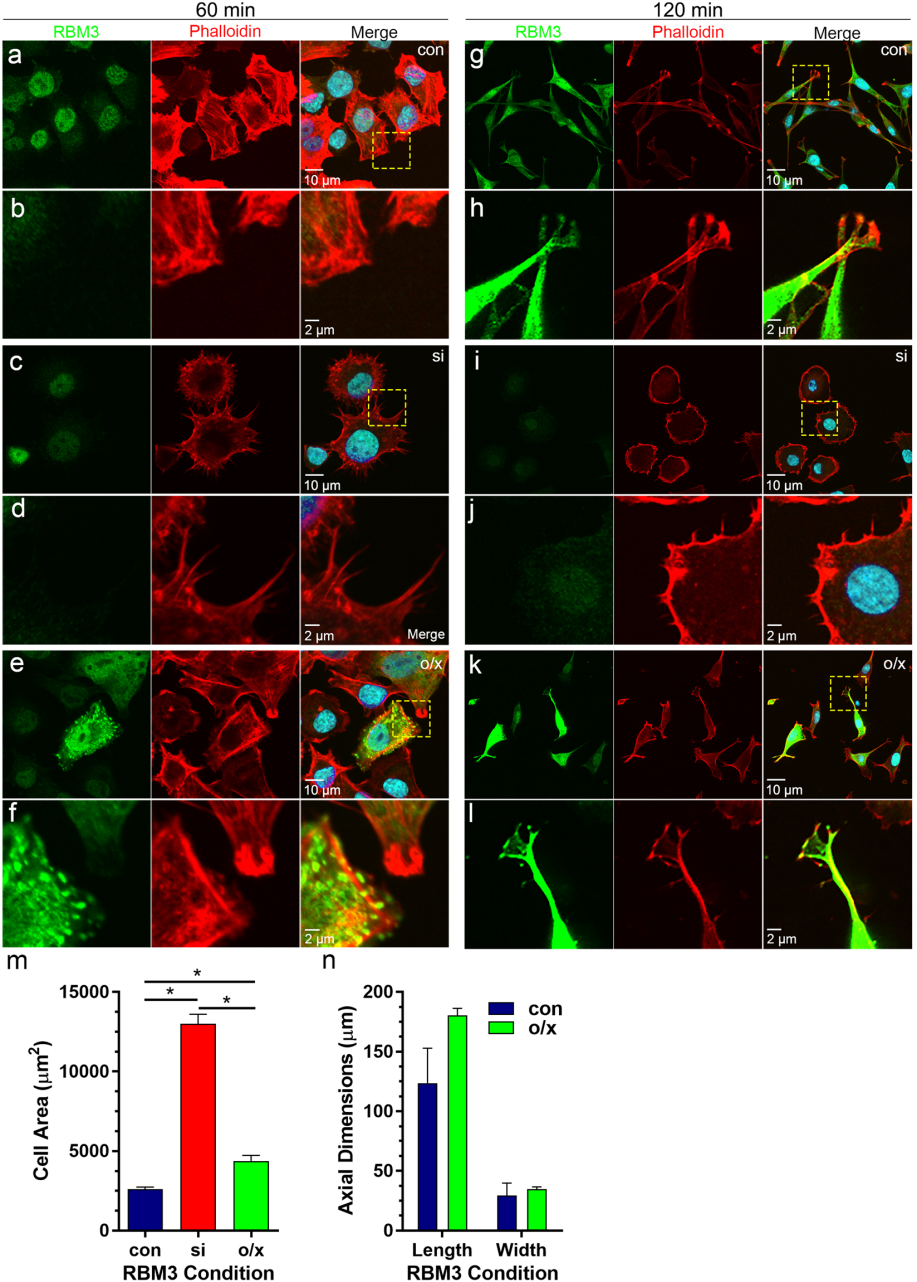
Perturbation of RBM3 expression alters cell spreading and morphology. B104 cells were mock transfected (con), transfected with an siRNA to knock down RBM3 (si) or transfected with a CMV-based expression vector to overexpress EYFP-RBM3 (o/x), then replated 48 hours later onto fibronectin-coated glass cover slips. Cells were fixed at 60 minutes (**a–f**) and 120 minutes (**g–l**) post plating for imaging of RBM3 (green), F-actin (red, phalloidin), and nuclei (blue, DAPI). (**a,c,e**) Images of RBM3 and F-actin in B104 cells under control (**a**), RBM3 knockdown (**c**), and RBM3 overexpression (**e**) conditions at 60 minutes post plating. (**b,d,f**) Close-up images of areas delimited by hatched yellow boxes in panels a, c, and e. Whereas control and RBM3-overexpressing cells exhibit the beginnings of cell polarity, RBM3 knockdown cells adopted a highly rounded or polygonal morphology with prominent microspikes. (**g,i,k**) images of RBM3 and F-actin in B104 cells plated as in panels, a,c,e, but fixed at 120 minutes. At this timepoint, cells lacking RBM3 adopt markedly different shapes than control and RBM3-overexpressing cells. (**h,j,l**) close-ups of areas delimited within panels g, i, and k. Whereas control and RBM3-overexpressing cells were elongated in a polar morphology with growth cone-like protrusions containing RBM3 and F-actin, cells lacking RBM3 did not develop polarity, becoming rounded and highly spread. RBM3 overexpressing cells had a more monopolar appearance compared to control cells, but with similar growth cone-like protrusions containing F-actin and RBM3. (**m**) Bar graph of cell area measurements in control (con; n = 32), RBM3 knockdown (si; n = 30), and RBM3 overexpression (o/x; n = 18) conditions (*p < 0.0001, unpaired t-tailed t-tests). (**n**) bar graph summarizing the maximum lengths and widths of cells in control vs RBM3 overexpression conditions; group data were not significantly different.

We tested the role of RBM3 in cell spreading by manipulating its expression in B104 cells, followed by replating and imaging of cell morphology, F-actin organization, and vinculin localization. B104 cells were transfected either with siRNAs to knockdown RBM3, or with an expression construct encoding EYFP-RBM3 as we have done previously^8^ (Supplementary Fig. S2). At 48 hrs post transfection, B104 cells were lifted by trypsinization and replated onto glass coverslips coated with fibronectin (10 μg/ml), then fixed and stained for RBM3, F-actin, and vinculin at 30, 60, and 120 minutes post plating. SICs still formed at 30 minutes in cells lacking or overexpressing RBM3 as evidenced by imaging of vinculin distribution (Supplementary Fig. S3B). However, subsequent spreading and cell morphology were dramatically altered by these manipulations (Fig. 2; Supplementary Fig. S3). At 60 minutes post plating, mock-transfected control B104 cells (Fig. 2a,b) and those overexpressing RBM3 (Fig. 2e,f) began to elongate into a bipolar/multipolar morphology, extending protrusions containing F-actin and RBM3, as well as vinculin (Supplementary Fig. S3A,C). However, cells in which RBM3 was knocked down adopted a non-polarized shape and were typically rounded or polygonal with prominent F-actin-rich microspikes (Fig. 2c,d; Supplementary Fig. S3B,E).

To further investigate the effects of RBM3 knockdown and overexpression on cell spreading, we imaged B104 cells in all RBM3 expression conditions at 120 minutes after replating when morphological elaboration of processes in control B104 cells is near complete. Differences in cell morphology between control, RBM3 knockdown, and RBM3 overexpression conditions were accentuated at this time point. Control B104s exhibited an elongated, multipolar morphology with growth cone-like F-actin-rich protrusions that contained RBM3, and local stress fibers at the poles (Fig. 2g,h). However, cells lacking RBM3 (Fig. 2i,j) were still rounded or polygonal with a much greater somatic diameter, exhibiting a ~400% increase in total cell area compared to controls (Fig. 2m). These RBM3 siRNA-induced changes in cell shape/polarity were rescued by co-transfection with an EYFP-RBM3 construct (Supplementary Fig. S4A); moreover, cells in which siRNA-mediated knockdown of RBM3 was inefficient were able to attain a polarized state (Supplementary Fig. S4B). B104 cells lacking RBM3 were also notable for the presence of a continuous ring of F-actin at the membrane that was punctuated by F-actin microspikes (Fig. 2i,j). In addition, the distribution of vinculin was altered such that it formed a discontinuous ring around the cells (Supplementary Fig. S3E) as foci that resembled focal adhesions (which are normally evident near the poles of elongating B104 cells). B104 cells overexpressing EYFP-RBM3 (Fig. 2k,l) adopted a more monopolar appearance, with growth cone-like F-actin-rich protrusions that contained EYFP-RBM3; in addition, they exhibited a larger overall cell area than control cells (Fig. 2m) but did not differ significantly from control cells in long axis diameter or width (Fig. 2n). In both control and RBM3 overexpressing cells, RBM3 was distributed to the cytoplasm (including cell protrusions) and nucleus at 60 minutes and 120 minutes post plating (Fig. 2, Supplementary Fig. S3F). Taken together, these data indicate that RBM3 regulates cell spreading and the development of polarity, potentially through early effects at the SIC stage where it may regulate translation events critical to cytoskeletal organization and adhesion.

### RBM3 promotes cell migration and regulates migration mode

**Figure 3:**
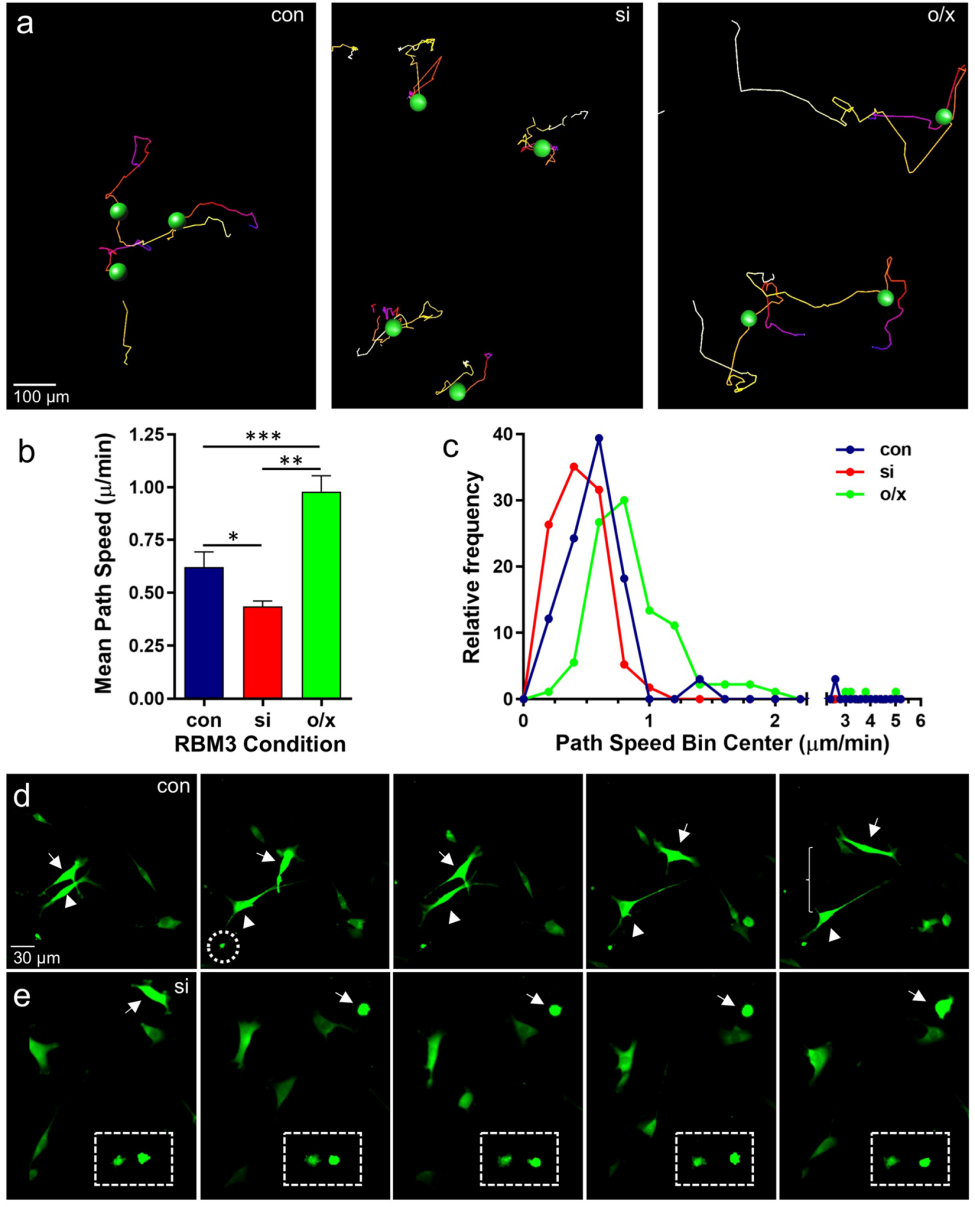
RBM3 promotes migration and regulates migration mode. B104 cells were transfected with EGFP alone (con), EGFP plus RBM3 siRNA to knockdown RBM3 (si), or a construct encoding an EYFP-RBM3 fusion protein (o/x). After 48 hrs, cells were replated onto fibronectin-coated coverslips and transferred to a homeostatic chamber for live imaging of cell movement. **(a)** Representative images of cell migration paths during live imaging of EGFP and EYFP-labeled B104 cells in the three treatment conditions. Path color indicates temporal progression: start = white; end = red. (**b**) Graph of the average path speeds displayed by B104 cells in the three treatment conditions (n = 33, con; n = 57, si; n = 90, o/x; *p < 0.05, **p < 0.0001; ***p < 0.001 2-tailed t-tests). (**c**) Relative frequency distribution of path speeds in the three treatment conditions; knockdown and overexpression of RBM3 significantly skewed the distribution of path speeds (con vs si, p < 0.05; con vs o/x, p < 0.0005; si vs o/x, p < 0.0005, Kolmogorov-Smirnov test). (**d**) Frames from Supplementary Movie S2 showing filopodia-based, mesenchymal–like migration of highly polarized cells in the control condition (con). Long extensions with growth cone-like ends (hatched circle) are evident in the control (and RBM3 overexpression) condition, but not after RBM3 knockdown (si). Two highly polarized cells (arrowhead and arrow) are seen migrating away from each other (**e**) Frames from Supplementary Movie S5 illustrating the morphology of cells in the knockdown condition as they migrate; some remain rounded (hatched rectangle) with continued blebbing. Another cell can be seen switching from a slightly elongated state with short protrusions, to a rounded, blebbing state (arrow).

The prominent effects of RBM3 on cell spreading and its localization to cell protrusions where adhesion and cytoskeletal assemblies are coupled to facilitate directional movement suggest that RBM3 also regulates cell migration. This would be consistent with findings that RBM3 is highly expressed in migrating neurons of the developing and adult rodent brain^28^, and in long filopodial extensions of invasive breast cancer cells^30^. To test this possibility, we manipulated RBM3 expression in B104 cells and then monitored cell movement in real-time imaging experiments. 48 hrs after manipulation of RBM3 expression, B104 cells were replated onto coverslips coated with 5 μg/ml fibronectin (a lower concentration than used in spreading assays) and transferred to a homeostatic chamber (35–37 °C + 5% CO_2_) mounted onto a Zeiss Radiance 2100 confocal microscope for time lapsed imaging. The movement of individual cells expressing EGFP in control and RBM3 knockdown conditions, and cells expressing EYFP-RBM3 (RBM3 overexpression), was quantified with automated cell tracking tools in Imaris software (Bitplane). Representative cell tracking experiments are shown in Fig. 3a, with cell movement tracks indicated by colored lines; examples of real-time imaging are provided in Supplementary Movies 1–7. The average migration rate of B104 cells was significantly reduced by knockdown of RBM3, and greatly enhanced by overexpression of RBM3 (Fig. 3b). These effects are evident in frequency distributions of path speeds (Fig. 3c) – *i.e*. all rates calculated from serial image captures – in which it can be seen that higher RBM3 expression significantly skews the distribution towards faster migration speeds, whereas lower RBM3 expression significantly skews towards lower speeds.

Interestingly, changes in RBM3 expression status also affected the mode of migration. Control B104 cells and those overexpressing EYFP-RBM3 exhibited mesenchymal-like migration, extending long, thin processes from a highly polarized state (Fig. 3d; Supplementary Movies S1, S2, S6, S7), whereas cells in which RBM3 was knocked down were more rounded and appeared to use a combination of mechanisms involving much shorter lamellipodia-like extensions and blebbing (Fig. 3e; Supplementary Movies S3–S5). Moreover, cells lacking RBM3 appeared to have less directional migration in that their paths were shorter and more random compared to control and RBM3 overexpressing cells that had long migration tracks. Some cells in the RBM3 knockdown condition did not migrate at all, remaining circular and continuing to form SIC-like blebs (see Fig. 3e and Supplementary Movie S5), while others switched from a partially elongated state with short protrusions to a rounded, blebbing morphology (Fig. 3e, Supplementary Movies S3–S4). These differences in migration mode resembled the distinct migratory modes seen in many cancer cells that undergo mesenchymal to amoeboid (MAT) and amoeboid to mesenchymal (AMT) transitions during metastasis^48–53^. Migration in mesenchymal states involves extension of long processes (as seen in control and RBM3 overexpressing cells) and extracellular proteolysis; amoeboid migration involves blebbing on cells that are more circular (as seen after RBM3 knockdown).

### RBM3 promotes wound healing

**Figure 4:**
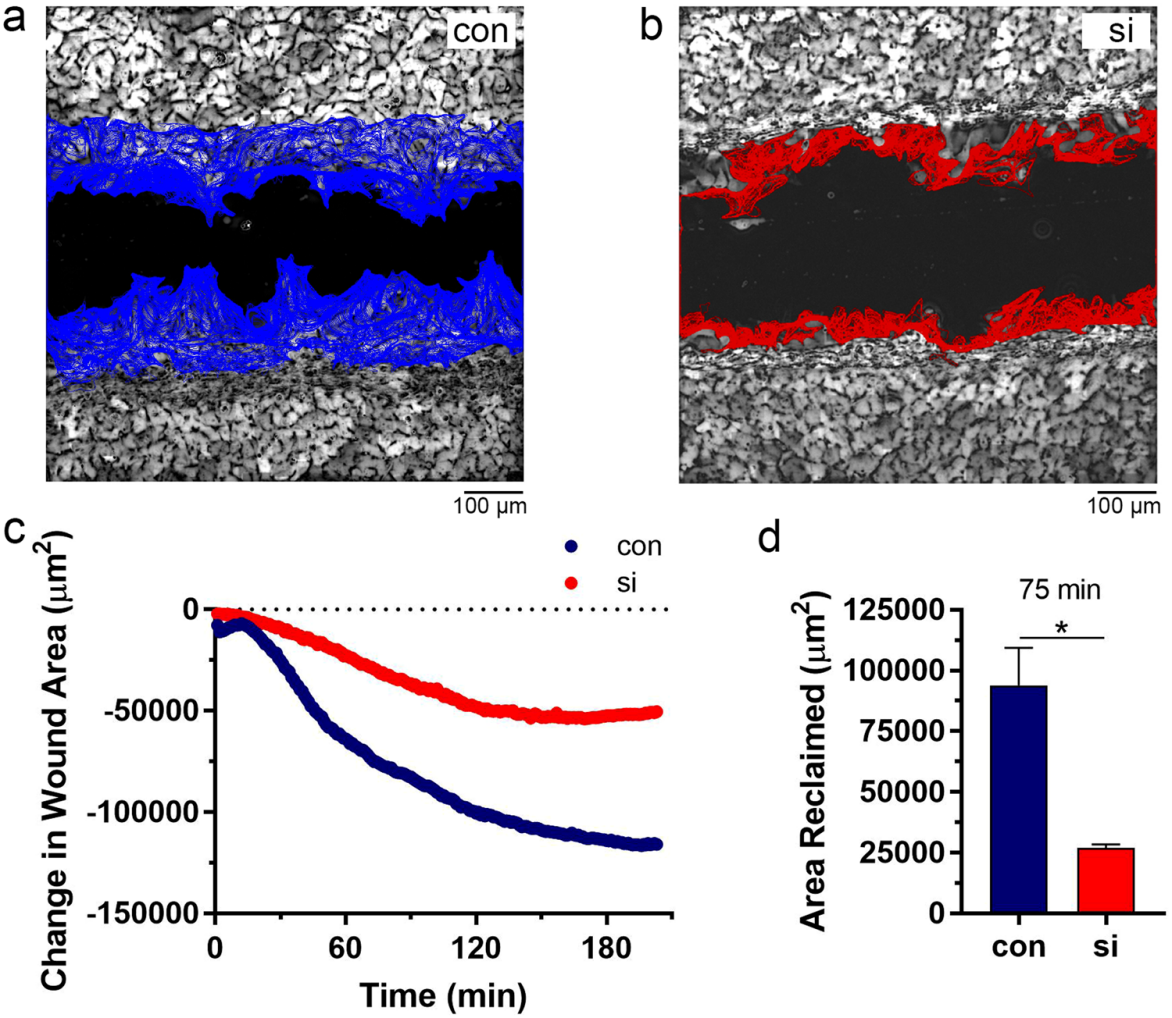
Reduction of RBM3 impairs wound healing. Scratch wounds were created in confluent cultures of 3T3 fibroblasts and wound closure was imaged in real time under homeostatic conditions. (**a** and **b**) Representative images of wounds and superimposed traces of fibroblast migration into the wound area in control (**a**, blue) and RBM3 knockdown (**b**, red) conditions. (**c**) Graph of the extend of wound closure as a function of time in the control (con) and RBM3 knockdown (si) experiments shown in panels a and b. (**d**) Graph summarizing group data on wound closure in terms of area reclaimed, normalized to scratch length, in control and RBM3 knockdown conditions (con, n = 4; si, n = 3; *p < 0.05 1-tailed t-test).

The above data indicated a role for RBM3 in directional migration. As another approach to evaluating the role of RBM3 in directional cell migration, we tested the effects of perturbating RBM3 expression in an *in vitro* wound healing assay using NIH3T3 fibroblasts. In confluent cultures of 3T3 cells grown on fibronectin-coated glass coverslips, a “wound” was created by dragging the edge of a toothpick across the coverslip to create a linear cell-free gap of approximately 300 μm in width. Live imaging was used to monitor the rate at which this gap was closed by migrating 3T3 cells. Automated analysis of these movies (Image-Pro Plus software) revealed that wound healing was greatly impaired by knockdown of RBM3 (Fig. 4a vs b and Supplementary Movies S8, S9). Over time, large and statistically significant differences in wound area became evident in RBM3 knockdown vs control cells. This is illustrated in a time plot of wound area (Fig. 4c), and in a graph of the area reclaimed at 75 minutes after creation of the wound (Fig. 4d).

### RBM3 is present in SICs and blebs of primary myoblasts and regulates myotube formation

Migrating cells *in vitro* and *in vivo* can form a variety of cell protrusion types, including actin-polymerization-driven lamellipodia and filopodia, and contractility-driven blebs. As an additional cell model in which to study the role of RBM3 in cell migration, we utilized primary skeletal muscle myoblasts in which migration is mediated by directional blebbing^54^. This system also allows assessment of the functional significance of factors regulating migration in terms of the physiologically relevant process of myotube formation, which depends on directional migration and fusion of myoblasts.

**Figure 5:**
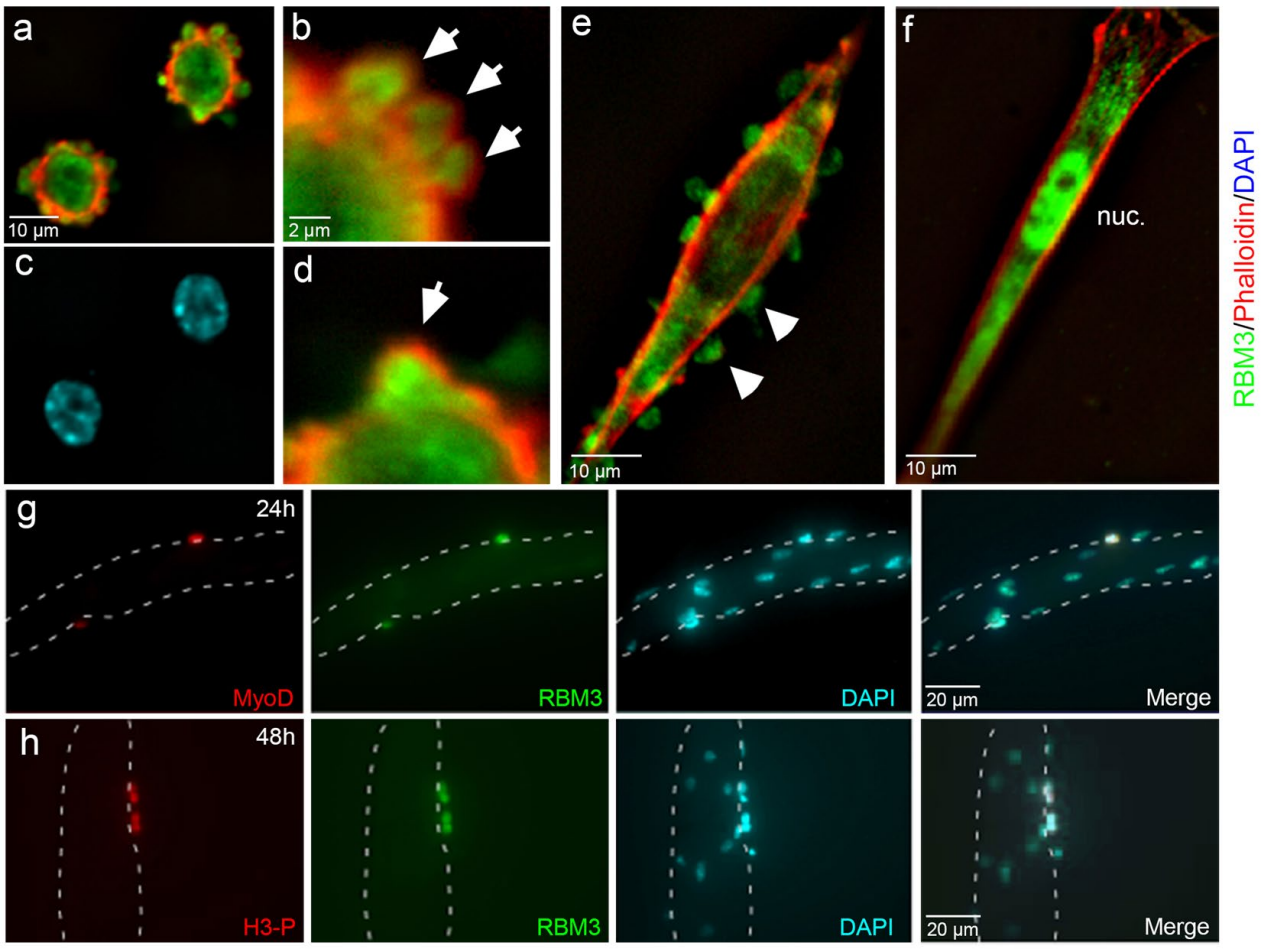
Dynamic localization of RBM3 in differentiating primary myoblasts. (**a**) Image of F-actin (red, phalloidin), RBM3 (green) in myoblasts fixed shortly after plating (1 hr) onto fibronectin. SICs are clearly visible along the edges of recently adherent cells as F-actin-delimited outpouchings of membrane. RBM3 is concentrated within SICs. (**c**) DAPI-stained nuclei in cells from Panel a. (**b** and **d**) Blowup of upper and lower margins of cell from the top-right portion of panel A. SICs (arrowheads) can be seen clearly with a peripheral F-actin ring surrounding an RBM3-rich core. (**e**) Image of a myoblasts partially differentiated into a myotube. At this stage, RBM3 is present in membranous blebs devoid of F-actin that are known to mediate myoblast migration. (**f**) Image of a fully differentiated satellite cell. RBM3 relocalizes from membrane protrusions to the cytoplasm and nucleus (nuc). (**g**) Images of a myotube explant at 24 hrs in culture in which activated satellite cells migrating along the myotube border (dashed outline) co-express MyoD (red) and RBM3 (green). (**h**) Images of dividing satellite cells along the border of a myotube that co-label for histone H3 phosphate (H3-P, red) and RBM3 (green).

We characterized RBM3 distribution and F-actin organization in myoblasts plated onto collagen. When cultured in growth medium with 20% serum and basic fibroblast growth factor (bFGF), primary skeletal myoblasts remain in a highly proliferative state for several passages. Stimulation of differentiation by serum withdrawal induced rapid changes in cell shape and remodeling of the actin cytoskeleton as observed by phalloidin staining for F-actin (Fig. 5); these cells were positive for myogenin, confirming that myoblasts were starting their differentiation process into myotubes (Supplementary Fig. S5). At early time points following serum withdrawal, SICs were visible along the periphery of adherent myoblasts (Fig. 5a–d), exhibiting the characteristic F-actin-ringed shape. RBM3 was concentrated within these SICs (Fig. 5a,b,d and Supplementary Fig. S6A). Subsequent imaging of myoblasts as they differentiated into myotubes revealed a distinct localization of RBM3. Differentiating myoblasts adopted an elongated shape with a prominent membrane F-actin cytoskeleton. At this stage, RBM3 was localized in the cytoplasm in a granule-like pattern and in a series of membranous outpouchings (blebs) that, unlike SICs seen shortly after plating under differentiating conditions, were not surrounded by F-actin (Fig. 5e); very little staining was seen in nuclei. These blebs are critical to the directional migration of myoblasts and their subsequent fusion into myotubes in response to injury^54–56^. As myoblasts differentiated further, they became highly elongated without blebs. At this stage, RBM3 was localized to the cytoplasm and nucleus (Fig. 5f, Supplementary Fig. S7). In culture, multinucleated myotubes form that show lateral lamellae (containing G-actin), as well as myotube flattened ends (containing F-actin) that are attached to substrate^54^. In these myotubes, RBM3 was differentially localized to the cytoplasm in the middle of myotubes, and in nuclei near the flattened ends (Supplementary Fig. S7). Thus, RBM3 exhibited dynamic localization in myoblasts from initial activation (Fig. 5, Supplementary Fig. S7) to their assembly into multinucleated myotubes.

*In vivo*, myoblasts are derived from satellite cells, muscle-specific stem cells involved in muscle regeneration^57–59^, which migrate along myofibers as part of the regenerative process. To characterize RBM3 expression in activated satellite cells, we used floating single myofiber cultures^60^. In these cultures, satellite cells are maintained beneath the basal lamina and have virtually no other influences than the myofiber environment. In serum–rich medium, satellite cells activate, migrate to the myofiber surface, and start to express MyoD and differentiate, mimicking the *in vivo* regenerative process^60^. We found that in single myofiber cultures RBM3 was highly expressed in migrating, MyoD-positive satellite cells after activation and differentiation (Fig. 5g, Supplementary Fig. S6B), including those undergoing mitosis as marked by immunolabeling for phosphorylated histone H3 (H3-P, Fig. 5h, Supplementary Fig. S6B). Satellite cells in an activated state are known to migrate along myotubes before their incorporation by fusion, and RBM3 was seen in fusing satellite cells as well (Supplementary Fig. S7). We also detected expression of RBM3 in the nuclei and cytoplasm of human myoblasts and myotubes formed in culture (Supplementary Fig. S8).

**Figure 6:**
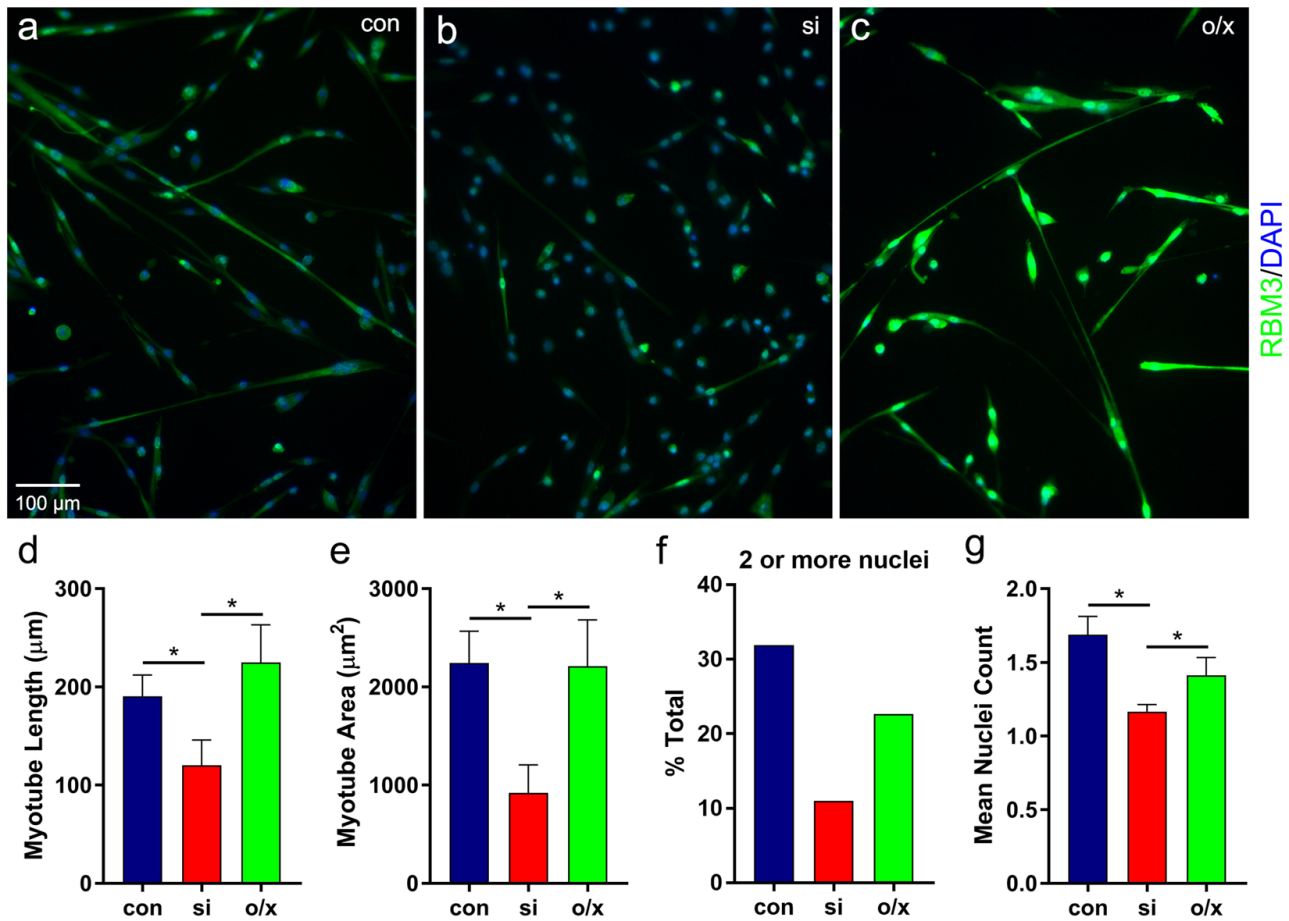
RBM3 regulates myotube formation *in vitro*. Satellite cells maintained under differentiating conditions were transfected with RBM3 siRNA (si), an EYFP-RBM3 expression construct (o/x), or subjected to mock transfection (con). (**a**–**c**) Images of myoblasts in the indicated RBM3 expression conditions. siRNA-mediated knockdown of RBM3 (**b**) impaired myotube formation from differentiating cells, whereas RBM3 overexpression (**c**) enhanced it. (**d–g**) Graphs summarizing the length (**d**) and area (**e**) of formed myotubes, the percentage of all cells incorporated into myotubes containing at least 2 nuclei (mean ± SEM; n = 43, 14, 17 for con, si, o/x, respectively; *p < 0.05, 1-tailed t-test) (**f**) and the mean nuclei count (**g**) of all cells in each RBM3 expression condition (n = 135, 127, 43 for con, si, o/x, respectively; *p < 0.05, 1-tailed t-test).

The localization of RBM3 in myoblast blebs and migrating satellite cells suggested that RBM3 regulates the process of bleb-based directional migration that is an essential step in muscle repair by activated satellite cells^54–56,61–63^. Thus, we studied the effects of manipulating RBM3 expression on myotube formation in cultures of primary skeletal myoblasts (Fig. 6). Knockdown of RBM3 in myoblasts (Fig. 6b) impaired myotube formation as measured by myotube length, area, number of nuclei, and the percentage of total cells integrated into myotubes (Fig. 6d–g). In contrast, while overexpression of RBM3 (Fig. 6c) led to a slightly longer average myotube length, this change was not statistically significant. Overall, the effects of RBM3 on myotube formation, along with results obtained in the wound healing assay, provide evidence that RBM3 regulates directional cell migration in processes relevant to tissue repair.

### Regulatory effects of RBM3 on cell shape involve RhoA-ROCK and CRMP2 signaling

**Figure 7:**
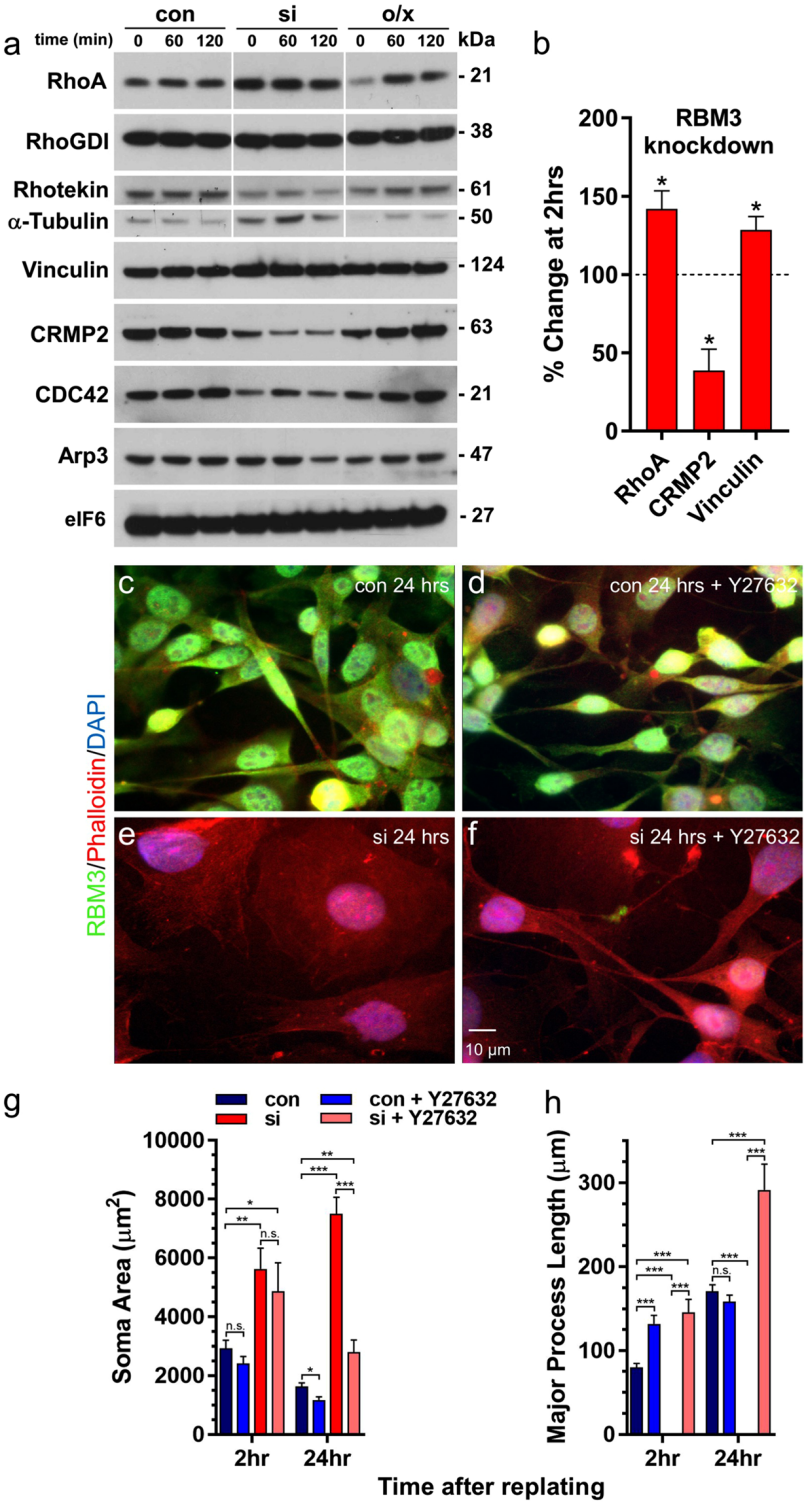
RBM3-regulated changes in cell morphology involve RhoA-ROCK signaling. (**a**) Western blots showing expression levels of the indicated proteins at 0, 60, and 120 minutes after replating of B104 cells maintained in control (con), RBM3 knockdown (si), and RBM3 overexpression (o/x) conditions. A large increase in RhoA is observed at all timepoints in RBM3 knockdown cells, along with tubulin and a modest increase in vinculin, an SIC component. CRMP2, rhotekin, and CDC42 were downregulated in the knockdown condition. In cells overexpressing RBM3, RhoA was initially downregulated. (**b**) Bar graph of changes in RhoA, CRMP2 and vinculin at 2 hrs in RBM3 knockdown vs control cells (n = 4 experiments; *p < 0.05, 1-sample t-test), normalized to eIF6 (present blots) or β-actin (additional experiments, Supplementary Fig. S11). (**c–f**) Images of RBM3 (green), F-actin (red, phalloidin), and nuclei (blue) in B104 cells under control (con) and RBM3 knockdown conditions (si), with and without the ROCK inhibitor Y27632 (100 μM). Inhibition of ROCK rescued cell polarity in RBM3 knockdown B104 cells (scale bar = 10 μm). (**g**,**h**) Graphs summarizing cell body areas (**g**) and major process lengths (**h**) at 2 and 24 hrs post replating in the following treatment conditions: control cells (con: n = 17 at 2 hrs; n = 23 at 24 hrs), RBM3 knockdown cells (si: n = 22 at 2 hrs; n = 20 at 24 hrs), control cells treated with Y27632 (con + Y27632: n = 15 at 2 hrs; n = 25 at 24 hrs), RBM3 knockdown cells treated with Y27632 (si + Y27632: n = 12 at 2 hrs; n = 15 at 24 hrs; *p < 0.05, **p < 0.005, ***p < 0.0005; t-tailed t-test). Cells in which RBM3 was knocked down, but not treated with Y27632, did not have processes to measure.

The mechanisms by which RBM3 regulates cell morphology could be highly complex given that it has a broad and diverse mRNA target pool, many known protein-protein interactions, and it stimulates global translation^5^. However, the nature of RBM3’s effects on cell polarity, spreading and directional migration provide phenotypic clues to the identity of factors involved. We observed that B104 cells under control and RBM3 overexpression conditions have mesenchymal characteristics, such as a polarized shape, migration by extension of long protrusions, and expression of vimentin (Supplementary Fig. S9). Knockdown of RBM3 resulted in a loss of cell polarity and exaggerated spreading. Moreover, knockdown of RBM3 – under conditions in which RBM3 is stably depleted for 24hrs or more (Supplementary Fig. S2) – impaired the ability of migrating B104 cells to extend long protrusions and switched their mode of migration to a non-directional, amoeboid-like mode. RhoA signaling is upregulated in cells transitioning from a polarized mesenchymal state to a rounded amoeboid-like morphology^48,51^, and both loss of polarity and amoeboid migration are mediated by Rho kinase (ROCK) activation downstream of RhoA^48,49,51,53,64,65^. Thus, we assessed whether manipulation of RBM3 levels alters the expression of RhoA and other factors during cell spreading and the establishment of polarity. We used a replating assay in which cells lacking or overexpressing RBM3 (Supplementary Fig. S2), and controls, were harvested for Western blot analyses at 0, 60 and 120 minutes after re-plating onto fibronectin. We found that, relative to control cells, RhoA protein was significantly upregulated in the RBM3 knockdown condition at all timepoints examined (Fig. 7a,b; Supplementary Fig. S10 and Supplementary Fig. S11), whereas it was initially downregulated in cells overexpressing RBM3. The SIC and focal adhesion component vinculin exhibited small, but consistent, upregulation after RBM3 knockdown (Fig. 7b). In addition to rounding, cells lacking RBM3 manifest microspikes at later stages of cell spreading (60–120 minutes), a morphological feature typically associated with CDC42 signaling. However, CDC42 levels were downregulated in this condition, and Arp3 (a downstream effector of CDC42 involved the generation of branched F-actin networks) was modestly downregulated at 120 minutes post plating.

An effector of RhoA signaling via ROCK, the collapsin response mediator protein 2 (CRMP2 or DPYSL2), was also reduced (>50%, Fig. 7b) after RBM3 knockdown. CRMP2 is a microtubule-binding protein that regulates neural polarity, Rho family GTPases^66–68^, and the collapse of growth cones in response to extrinsic cues such as myelin associated glycoprotein (MAG) and Nogo-66. Phosphorylation of CRMP2 by ROCK in response to MAG and Nogo-66 reduces microtubules and stimulates growth cone collapse^69,70^. This effect is phenocopied by knockdown of CRMP2^69^, which suggests that the upregulation of RhoA and downregulation of CRMP2 in cells lacking RBM3 have convergent, synergistic effects in the same pathway – *i.e*. both reduce CRMP2 function. No change was seen in an inhibitor of GDP dissociation from Rho family GTPases, RhoGDI (ARHGDIA), that regulates RhoA activation by MAG, but a decrease in the expression of the RhoA scaffolding protein rhotekin was seen with RBM3 knockdown. Levels of tubulin were upregulated. No changes were evident in the eukaryotic initiation factor 6 (eIF6, Fig. 7a) and β-actin (Supplementary Fig. S11), which were used to normalize values of RhoA, CRMP2 and Vinculin presented in Fig. 7b.

**Figure 8:**
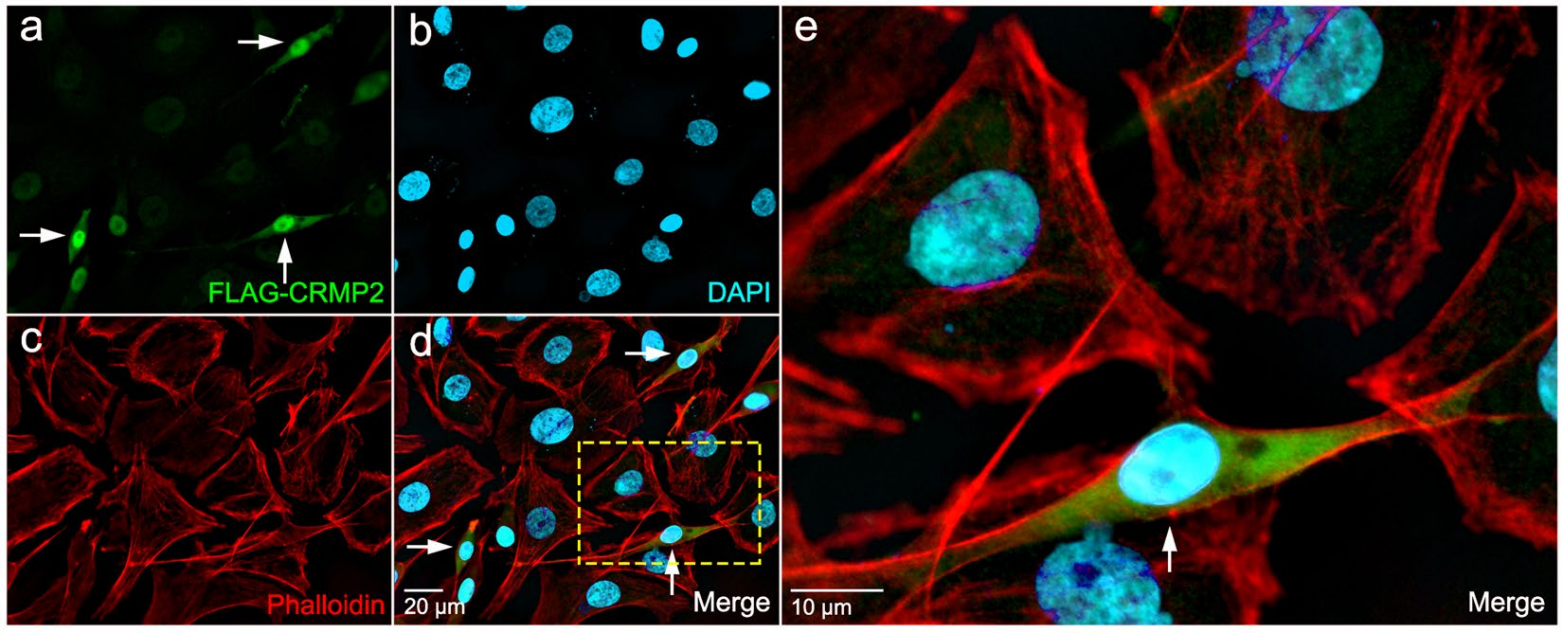
RBM3-associated changes in cell morphology involve CRMP2, a substrate of ROCK. (**a–d**) Images showing rescue of cell polarity and spreading in RBM3 knockdown B104 cells by transfection with a FLAG-CRMP2 construct: (**a**) FLAG-CRMP2 (green); (**b**) nuclei (DAPI, blue); (**c**) F-actin, (phalloidin, red); (**d**) overlay. (**e**) Enlargement of area highlighted by yellow box in **d**. Arrows in panels a, d and e show FLAG-CRMP2 expressing B104 cells, which have rescued polarity.

Based on our results, we hypothesized that RhoA-ROCK and CRMP2 signaling contribute significantly to RBM3-mediated changes in cell shape. To test this idea, we incubated control and RBM3 knockdown B104 cells with the ROCK inhibitor Y27632 (100 μM) from 30 minutes before to 24 hrs after replating cells onto fibronectin-coated coverslips. Cells were fixed at 2 hrs and 24 hrs post plating for imaging of F-actin (phalloidin) and RBM3. As seen earlier, control cells exhibited a highly polarized morphology at 2 hrs; this general morphology was maintained for 24 hrs and did not change with addition of the ROCK inhibitor (Fig. 7c,d). Cells lacking RBM3 exhibited the characteristic rounded or polygonal shape at 2 hrs and 24 hrs without Y27632, but adopted a highly polarized shape when incubated with the ROCK inhibitor (Fig. 7e,f). Thus, ROCK inhibition rescued the loss of polarity induced by knockdown of RBM3. However, while ROCK inhibition rescued polarity in cells lacking RBM3, the cell bodies of many cells in this condition appeared more spread out than in control cells, and neurites appeared to be longer. Indeed, at 2 hrs and 24 hrs after plating, neurites in RBM3 knockdown cells treated with Y27632 were longer and cell bodies remained more spread than in control cells (Fig. 7d,f,g). This suggested that even though polarity was rescued by ROCK inhibition, molecular changes conferring exaggerated spreading may still have been present and manifest in both enhanced process outgrowth and somatic area. Consistent with the idea that changes in RhoA and CRMP2 after RBM3 knockdown have similar influences in a common RhoA-ROCK-CRMP2 pathway, polarity was also rescued by overexpression of CRMP2 (Fig. 8).

### A model of RBM3’s influence on cell spreading and migration

**Figure 9:**
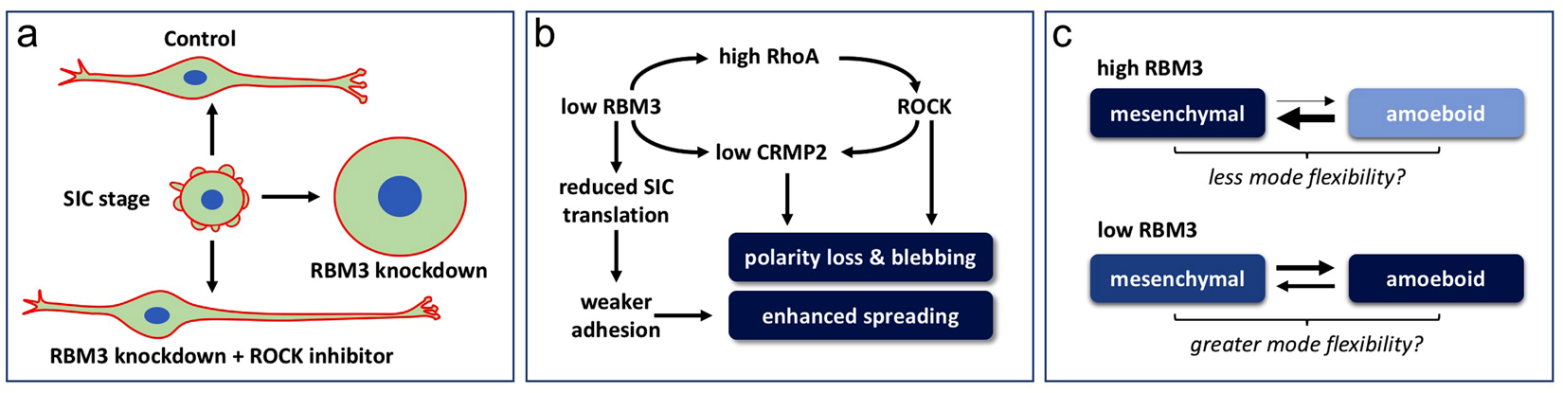
Model of RBM3’s influences on cell morphology and migration. (**a**) Schematic of the morphological trajectories taken by B104 cells under control, RBM3 knockdown, and RBM3 knockdown plus ROCK inhibitor conditions. Replated B104 cells initially exhibit SICs and a rounded morphology. Whereas control cells go on to adopt a bi- or multi-polar shape, cells lacking RBM3 lose polarity and undergo exaggerated spreading. Inhibition of ROCK during replating of RBM3 knockdown cells rescues polarity but enhances process elongation. (**b**) Diagram of proposed mechanisms by which RBM3 regulates cell polarity and spreading. Knockdown of RBM3 results in parallel changes in translation and a RhoA-ROCK-CRMP2 pathway. Elevated RhoA expression activates ROCK, a kinase involved in the transition from a mesenchymal to an amoeboid state. Reduced levels of CRMP2, a microtubule binding protein that is inhibited by ROCK phosphorylation, contributes to loss of polarity. (**c**) Schematic of hypothesized effects of RBM3 expression level on transitions between mesenchymal and amoeboid states. High levels of RBM3 lock cells into a highly polar, mesenchymal like mode of migration. Low RBM3 may favor an amoeboid state, but still be permissive to transitions between amoeboid and mesenchymal modes of migration, so-called MAT and AMT, respectively. This is may enhance metastasis of cancers by enhancing migration mode flexibility.

Taken together, our data indicate that RBM3 regulates cell polarity, spreading, and migration. Mechanistically, this is in part due to effects on RhoA-ROCK signaling and CRMP2, and it is likely that modulation of translation is also involved. A proposed model of these morphoregulatory effects is shown in Fig. 9. RBM3 is present in SICs formed during initial stages of spreading, and its levels at the time of replating impact the morphological trajectory that cells take. Control cells and cells overexpressing RBM3 ultimately adopt a polarized shape, whereas cells lacking RBM3 lose polarity and become rounded and highly spread (Fig. 9a). The loss of polarity is rescued by ROCK inhibition (and CRMP2 overexpression), but cell extensions become longer in the RBM3 knockdown condition when ROCK is inhibited, perhaps reflecting the continued activity of processes underlying exaggerated spreading, but in the context of a polarized state. We hypothesize that these processes are translation-dependent and dissociable from RhoA-ROCK signaling changes involved in the loss of polarity (Fig. 9b). RBM3 also strongly regulates the rate and mode of migration. Control and RBM3 overexpressing cells migrated in a mesenchymal mode involving extension of long protrusions, while cells with near complete knockdown of RBM3 migrated via an amoeboid-like mode. We hypothesize that low (non-zero) levels of RBM3 are more permissive to transitions between these migration modes (Fig. 9c). The metastasis of many cancers is facilitated by migration mode flexibility – *i.e*. the ability to undergo MAT and AMT transitions – and we hypothesize that high RBM3 expression impairs this flexibility by “locking” cells into a mesenchymal mode. This would help explain the large number of reports linking high RBM3 expression to reduced metastasis and better clinical outcome across many cancers.

## Discussion

Our data show that RBM3 exerts a potent regulatory influence on cell polarity, spreading and migration. In multiple cell types, RBM3 was observed within SICs formed shortly after plating onto several substrates, and within cellular protrusions – such as growth cone-like extensions in neuroblastoma cells and membrane blebs in myoblasts – that developed during the course of cell spreading and migration. Manipulation of RBM3 expression had a profound effect on cell polarity, spreading and migration, and impacted the aggregate behavior of cells in the processes of myotube formation and wound healing that depend on directional migration. Rounding of cells after knockdown of RBM3 and an apparent switch in migratory mode suggested that RBM3 modulates Rho-ROCK pathway signaling. Indeed, the loss of cell polarity induced by RBM3 depletion was accompanied by upregulation of RhoA and downregulation of a ROCK effector, CRMP2, and it was rescued by a ROCK inhibitor and overexpression of CRMP2. These novel functions of RBM3 may be of significance in the contexts of tissue repair, metastasis, and development.

### The mechanistic basis of RBM3’s effects on cell polarity, morphology and migration

RBM3 has a large and diverse set of target mRNAs (E.D-V. unpublished observations) and protein-protein interactions, and it regulates miRNA biogenesis, all of which suggest that the influence of RBM3 on cell morphology is complex. Nevertheless, we obtained evidence that RhoA-ROCK-CRMP2 signaling is critical to RBM3’s effects on cell morphology. We observed that the B104 neuroblastoma cell line has mesenchymal characteristics, including a polarized morphology, expression of the epithelial-to-mesenchymal transition (EMT) marker vimentin, and migration in a manner typical of mesenchymal cells in which long protrusions are extended from a polarized state. Knockdown of RBM3 caused B104 cells to lose polarity upon replating and adopt a rounded, highly spread shape. Loss of cell polarity combined with the appearance of SIC-like blebbing are reminiscent of cells that have undergone MAT^32,48,49,51^. Another hallmark of MAT is elevated RhoA-ROCK signaling, which is essential for loss of polarity and the switch to an amoeboid migration mode^48,49,51,64^. RhoA levels were upregulated in RBM3 knockdown cells throughout the course of cell spreading, suggesting that elevated RhoA-ROCK signaling triggers the rounded, amoeboid morphology we observed in this condition. Accordingly, a ROCK inhibitor (Y27632) reverted the rounded B104 cells lacking RBM3 back to a mesenchymal like, polarized morphology. Interestingly, cellular protrusions that developed as a result of treating RBM3 knockdown cells with Y27632 were longer than in control conditions, suggesting that the mechanisms underlying loss of polarity and enhanced spreading are dissociable. Forced expression of CRMP2 (which was reduced by RBM3 knockdown) also reverted RBM3 deficient cells back to a polar morphology. As CRMP2 phosphorylation by ROCK inhibits microtubule polymerization^68^, this suggests that RBM3 has multiple effects on a RhoA-ROCK-CRMP2 pathway regulating cell polarity and morphology.

The exaggerated spreading in cells lacking RBM3 is likely due to changes in SIC translation events that regulate adhesion. At early stages of cell attachment and spreading, ~80% of total cellular translation is localized within SICs^32^, and inhibition of translation hinders the maturation of focal adhesions and cell adhesion to substrate^32^. Interestingly, the translation inhibitors anisomycin, puromycin, and cycloheximide have been shown to enhance the rate of cell spreading^71,72^ in 3T3 cells and chick embryo fibroblasts cultured in low serum, which may be related to a state of weakened adhesion to substrate^32,51^. Given that RBM3 enhances global translation^5^, loss of RBM3 may phenocopy the effects of translation inhibitors on cell spreading to some extent by reducing translation in SICs. We propose that loss of polarity and exaggerated spreading in cells lacking RBM3 reflects a combined effect of elevated RhoA-ROCK signaling and decreased global translation rate, respectively. Moreover, we postulate that enhanced process outgrowth in RBM3 depleted cells treated with ROCK inhibitor reflects the continuation of processes underlying increased spreading, but in the context of a polarized state. However, Mardakheh *et al*.^30^ recently demonstrated that cycloheximide at a concentration that enhances spreading in fibroblasts (10 μg/ml)^71^ destabilizes protrusions and inhibits mesenchymal morphology (inducing rounding) in the MDA-MB231 breast cancer cell line. This may indicate that knockdown of RBM3 either does not affect spreading by lowering translation, or that only modest decreases in translation promote spreading; this would be consistent with an inverted-U relationship between the degree of translational inhibition and spreading seen in fibroblasts^71^. Alternatively, loss of RBM3 may differentially affect the expression of specific cytoskeletal, adhesive, or other factors that lead to exaggerated spreading.

Changes in Rho-ROCK signaling and translation may also help to explain the effects of RBM3 perturbation on cell migration. Migration of B104 cells was greatly enhanced by overexpression of RBM3 and impaired by RBM3 knockdown. Control and RBM3 overexpressing cells displayed a mesenchymal-like mode of migration in which polarized cells extended long protrusions in the direction of migration. In contrast, the migration of cells with reduced RBM3 appeared amoeboid in that cells were more rounded, did not extend long protrusions, and exhibited blebbing. Fibroblasts, endothelial cells, and many types of cancer cells migrate in a mesenchymal mode that depends on Rac/CDC42 signaling and extracellular proteolysis^51^. However, many cancers and some somatic cells (*e.g*. myoblasts and satellite cells) can also enter an amoeboid migration mode that is dependent on RhoA-ROCK signaling and weaker adhesion, but lacks dependence on extracellular proteolysis^48,49,51,54,73,74^. In light of our findings, it is reasonable to speculate that high RBM3 expression promotes mesenchymal migration by stimulating local translation events required for the maturation of focal adhesions and the extension of long protrusions, whereas low RBM3 levels favor an amoeboid mode by increasing RhoA expression and impairing the maturation of strong adhesion complexes.

### RBM3 and wound healing: might RBM3 facilitate re-epithelialization?

We used a standard wound healing assay primarily to assess the effects of RBM3 on cell migration, but the results obtained may also bear on long-standing issues regarding the effect of temperature on wound healing. It is well established, particularly in the surgical setting, that the multifaceted process of wound healing is facilitated by heat (or, rather, the prevention of hypothermia), and that it is slowed during the application of hypothermia^75–77^. Yet, it is also clear that transient hypothermia can have beneficial effects on wound healing, such as reduction of inflammation and scar formation^77,78^. In the 3T3 cell scratch assay, we observed that wound closure was severely impaired by knockdown of RBM3. *In vivo*, fibroblasts migrate into wounds via a mesenchymal like mode and facilitate wound healing by reestablishing the extracellular matrix for keratinocytes. To the extent that the 3T3 scratch assay models this process, it suggests that induction of RBM3 by transient hypothermia might facilitate re-epithelialization. It is notable in this regard that hibernating black bears, unlike smaller hibernators, demonstrate highly efficient wound healing with reduced scarring^77^. The difference in wound healing abilities between bears and smaller hibernators may lie in the depth and temporal profile of torpid states: bears maintain mild to moderately hypothermic core body temperatures (30–35 °C) during hibernation with daily warming periods in which dermal temperatures approach euthermia^79^, whereas smaller hibernators such as ground squirrels can approach near freezing body temperatures^80^ and only warm during intra-hibernation arousals that occur every 10–20 days. It is generally held that the beneficial effects of torpor and transient hypothermia involve gene expression and signaling events triggered by cold^22,81,82^. In hibernating bears, a combination of mild hypothermia with frequent bouts of warming might be optimal for triggering RBM3 and other benefits of hypothermia without incurring the negative aspects of protracted cooling on wound healing, such as infection and reduced blood flow. If this is the case, then therapeutic induction of RBM3 during euthermia by pharmacological strategies, or by alternating episodes of mild hypothermia and euthermia, may facilitate re-epithelialization and realize some of the benefits of hypothermia (such as reduced cell death, which may also involve RBM3^20,22^), without risking its negative consequences. Based on observations that RBM3 negatively regulates the RhoA-ROCK pathway, some of the putative benefits of RBM3 on wound healing may be recapitulated by inhibition of RhoA-ROCK signaling, which has been shown to promote corneal wound healing^83,84^.

### RBM3 and the response of muscle to disuse, stress, and injury

Taken with our prior work, the effects of RBM3 on myoblasts and their assembly into myotubes suggest that RBM3 has pleiotropic roles in muscle preservation and regeneration. Previously, we showed that RBM3 protects cultured myoblasts from various cell stressors^20^ – reducing both necrosis and apoptosis – and may help preserve muscle mass in the context of disuse^19^. It is notable here that muscle mass is largely preserved during hibernation in black bears, a state of disuse and severe metabolic stress (starvation and hypothermia) in which RBM3 is highly upregulated in all tissues^27^. Mechanistically, the protective influence of RBM3 in muscle is most likely due to a combination of specific effects on apoptotic and atrophy factors via the miRNA pathway^8,85^, and the ability of RBM3 to stimulate global translation^5^. However, the present data suggest that RBM3 may also help preserve muscle by promoting the migration of cells involved in injury repair. Satellite cells are a resident stem cell population of muscle that are essential for muscle growth and regenerative capacity^57–59,86^. In the context of injury, satellite cells shift from a quiescent to an activated state in which they proliferate and differentiate into myoblasts^87^ that integrate into myotubes where needed to repair damaged muscle. We observed that RBM3 promotes myotube formation, and that it is present in activated satellite cells, and in SICs and blebs of differentiating and migrating myoblasts. Myoblasts migrate using both actin polymerization-driven lamellipodia formation and contractility-driven blebs^61–63^. Under the plasmalemma, satellite cells migrate using a lamellipodial mechanism. However, activated satellite cells and myoblasts switch to a blebbing-based motility that is necessary for their migration to sites of damage^54,55^ and for proper alignment of myoblasts for fusion into myotubes^55,56^. The enrichment of RBM3 in these blebs and the attenuation of myotube formation by RBM3 knockdown suggest that RBM3 may play an important role in muscle repair after injury by promoting migration of satellite cells and myoblasts. It will be of interest to determine if perturbation of RBM3 expression *in vivo* affects regenerative responses that depend on satellite cells. If this is the case, then it would place RBM3 as a nodal factor in muscle health, promoting both *(i)* the maintenance of muscle mass via cell protective and anabolic functions and *(ii)* local stem cell-based regenerative responses. It would also suggest that RBM3 presents a unique target for pharmacological enhancement of muscle maintenance and regeneration.

### RBM3 in the neurogenic niche

Previously, we reported that the expression of RBM3 in brain peaks during the early postnatal period, with the highest levels evident in areas reported to have high translation rates and in niches where neurogenesis and migration of newly formed neurons occur^28^. For example, RBM3 was strongly expressed in cells of the subventricular zone that co-expressed Ki67, and in nestin-positive cells migrating out from this region; RBM3 was also highly expressed in Ki67-positive and doublecortin-positive cells of the rostral migratory stream. High expression of RBM3 in these cells may have a dual purpose in promoting migration and in enhancing the biogenesis of microRNAs (*e.g*. let-7 family) involved in differentiation^8^. It will be of interest to determine if induction of RBM3 in neurogenic niches can facilitate the migration and morphological differentiation of newly formed neurons.

### Do the morphoregulatory functions of RBM3 inform on its role in cancer and utility as a biomarker?

Our finding that RBM3 promotes cell migration – a property that would be expected to enhance cancer metastasis – appears to be at odds with numerous reports that high RBM3 expression limits cancer progression. Moreover, the detection of RBM3 in invadopodia of MDA-MB231 cells^30^, a highly invasive breast cancer cell line with mesenchymal characteristics^88^, suggests that it may promote metastasis. In our studies, RBM3 localized to SICs and to protrusions in the B104 neuroblastoma cell line that are morphologically very similar to the invadopodia of MDA-MB231 cells. Overexpression of RBM3 markedly enhanced mesenchymal-like migration of B104 cells, whereas RBM3 knockdown impaired it. These findings suggest that RBM3 promotes migration of cancers that have undergone EMT. However, studies in metastatic melanoma and other cancers find that high RBM3 expression is associated with reduced invasiveness^29,89,90^. Accordingly, high nuclear (and in some cases cytosolic) expression of RBM3 is associated with improved disease free and overall survival in a wide range of cancers, including: melanoma, breast, prostate, epithelial ovarian, testicular, colorectal, urothelial bladder, esophageal and gastric, and astrocytoma^29,89–96^.

A potential explanation for this discrepancy is that high RBM3 expression in general (which is more easily detected and quantified in nuclei, as opposed to cell protrusions, in histological sections) restricts cancer cells to a mesenchymal mode of migration. Many cancers that undergo EMT (including melanoma) can rapidly transition from mesenchymal to amoeboid states (MAT) and back (AMT) in response to changing extracellular cues^32,48,49,51,53,74,97^. This flexibility in migration mode enhances net metastasis^32,52,53,98–100^. It has been shown that RhoA-ROCK signaling is essential for MAT, and that inhibition of this pathway reduces metastasis in melanoma, colorectal, and triple negative breast cancer^48,53,100^. Thus, it is plausible that lower RBM3 expression is permissive to MAT because it enhances RhoA-ROCK signaling and reduces translation events that support strong adhesion, whereas higher RBM3 “locks in” a mesenchymal mode by stimulating local translation. If so, then the metastasis of cancers that rely on MAT-AMT flexibility for migration might be hindered by upregulation RBM3. These ideas are potentially challenged, however, by studies in astrocytoma^101^ and erythroblastosis virus E26 oncogene homolog-positive prostate cancer^102^ (but see^29,90^), which show that nuclear expression of RBM3 is positively associated with higher grade cancers and predicts a poorer clinical outcome. Another way to reconcile RBM3’s effects on migration and its association with better cancer outcomes may be the subcellular distribution of RBM3. Most of the histological data on RBM3 in cancer show that high nuclear expression is associated with better clinical outcomes^29,89–96^. Cancers with high nuclear RBM3 might adopt this status as a result changes in nucleocytoplasmic transport that shunt RBM3 away from cell protrusions where it would otherwise enhance mesenchymal migration. However, several of these reports also find elevated RBM3 protein levels in the cytoplasm^29,89,92^.

A clearer picture of RBM3’s role in cancer will require an understanding of the composite influence of RBM3’s varied molecular functions. In the context of oncogenesis, these are thought to include *(i)* a protooncogene-like effect mediated by stabilization of AU-rich element-bearing mRNAs such as COX2 and VEGF^6^, *(ii)* modulation of checkpoint pathway proteins^103,104^ and GSK3β and β-catenin signaling^105^, and *(iii)* regulation of microRNA biogenesis^8^. With regard to the latter, we reported that over 60% of microRNAs detected in B104 cells were positively regulated by RBM3 at the Dicer step, especially let-7 family members which were completely dependent on RBM3 for maturation^8^. Let-7 microRNAs inhibit stemness and oncogenesis^106,107^, and it is plausible that their upregulation by RBM3 is a driving factor in the association of high RBM3 expression with reduced metastasis and better clinical outcomes^108,109^.

In summary, we have elucidated novel functions of RBM3 in the regulation of cell polarity, spreading and migration. In addition, we have identified novel influences of RBM3 on RhoA-ROCK pathway signaling and CRMP2 that underlie RBM3’s effects on cell polarity. Taken with prior work, these data suggest that RBM3 may be a critical player in regenerative responses, such as wound healing and muscle maintenance, where its parallel influences on cell protection and cell migration may support several aspects of tissue repair. Insofar as RBM3 knockdown appeared to recapitulate several aspects of MAT exhibited by mesenchymal cancer cells, our data may also help inform on the mechanistic significance of correlations between RBM3 expression level and both the metastatic potential and clinical outcome of many cancers. Pharmacological upregulation of RBM3 might be of broad utility in amplifying endogenous stress response mechanisms linked to cellular protection and repair in many tissues, and present a new strategy to regulating the metastatic behavior of many cancers.

## Materials and Methods

### Cell culture

B104 neuroblastoma, NIH 3T3, N2A and HeLa cell lines (ATCC) were cultured at 37 °C, 5% CO_2_ in Dulbecco’s modified Eagle’s medium (DMEM, Gibco) supplemented with 10% fetal bovine serum (FBS, Gibco).

### Cultures of primary myoblast and single myofibers

All studies involving the use of skeletal muscle tissue from mice were carried out in accordance with protocols approved by the TSRI institutional animal care and use committee (IACUC). Mice, were housed and cared for in a facility accredited by the Association for the Assessment and Accreditation of Laboratory Animal Care, International (AAALAC). Prior to tissue harvest, mice were first anesthetized by halothane, followed by decapitation. Cells were prepared from skeletal muscle of postnatal day 4–5 wild type mice as previously described^110^ and cultured on collagen-coated plates in DMEM/F10 with 20% FBS and 5 ng/ml bFGF. Differentiation was induced by transferring cells to DMEM supplemented with 2% horse serum. Cells were fixed with 2% paraformaldehyde (PFA) at various time points after induction of differentiation and then stained with monoclonal antibodies to SMA (clone 1A4; Sigma), our RBM3 antibody, rhodamine-conjugated phalloidin, or combinations thereof. Single optical sections or Z-series were obtained using a Zeiss 710 laser-scanning confocal microscope. Images were analyzed with IMARIS imaging software (Bitplane). Primary human myoblasts were grown in Hams F-10 that contained 20% FBS, penicillin and streptomycin at 37 °C in a humidified 5% CO_2_–95% air atmosphere incubator. Differentiation of myoblasts into myotubes was achieved with MEM supplemented with 2% FBS for 7 days.

Single myofibers were prepared from the extensor digitorium longus muscle from 6 week old mice as described previously^60,111^. Briefly, muscles were digested in tubes with collagenase solution in a shaking water bath at 35 °C for 60–70 minutes. To isolate single myofibers, digested muscle was placed in a Petri dish with warm DMEM supplemented with horse serum and then triturated repeatedly using the large diameter heat-polished Pasteur pipette (pre-rinsed with DMEM/horse serum medium). Single myofibers were collected with smaller diameter heat-polished Pasteur pipettes under the microscope and maintained as floating cultures in DMEM supplemented with 5% horse serum. Myofibers were fixed after 24, 36 and 48 hours in culture and stained with antibodies to RBM3, MyoD, and H3-P.

### Drug treatments

ROCK inhibitor Y-27632 (100 µM, Sigma) was added to cells 30 minutes prior to replating, and then kept in the media thereafter until fixation with 4% PFA.

### Transfections

RBM3 levels were knocked down in B104 cells and primary myoblasts by transfecting 20 nM of an siRNA duplex targeting RBM3 – sense 5′-CCUUCACAAACCCAGAGCATT-3′ antisense 5′-UGCUCUGGGUUUGUGAAGGTG-3′ – with RNAiMax (Life Technologies) according to manufacturer’s protocol. RBM3 was overexpressed by transfecting EYFP-RBM3, pcDNA3.1-myc-RBM3, or pcDNA3.1-RBM3^5,28^ with lipofectamine 2000 (Life Technologies) according to manufacturer’s protocol. Overexpression of EGFP (Amaxa) and CRMP2 (FLAG-CRMP2, a generous gift from Dr. Christine Hall (University College London^67^)) was achieved with the same protocol. For both knockdown and overexpression, cells were processed (for Western blotting or immunocytochemistry) 48 hours after transfection. NIH 3T3 fibroblasts and primary myoblasts were transfected with an Amaxa Nucleofector device (Lonza). Program A24 was used to transfect 1 × 10^6^ cells using 3 µg of EGFP (con), or siRBM3 duplex (si) in NIH 3T3 cells.

### Immunocytochemistry

Cell lines grown on glass cover slips were fixed with 4% PFA for 10 minutes, rinsed 3 times with 1x PBS, permeabilized with 0.1% Triton X-100 for 10 minutes and blocked for 30 minutes with 10% normal goat serum (NGS) in PBS, 0.1% Triton X-100. Cells were incubated with a primary antibody dilution in 1% NGS 0.1% Triton X-100 for 1 hour at room temperature or 4 °C overnight (RBM3 1:1000, made in-house^28^; FUS, 1:200, Bethyl; vinculin 1:200, ProSci; myogenin 1:200, Santa Cruz; Histone 3p 1:200, EMD-Millipore; CRMP2 1:500, Abcam; FLAG 1:1000, Sigma; Vimentin 1:500, Sigma) and rinsed 3 times with PBS 0.1%- Triton X-100. For the secondary antibody, Alexa Fluor 488 goat anti-rabbit or Alexa Fluor 594 goat anti-mouse secondary antibody (Life Technologies) was diluted at a 1:1000 dilution and incubated for 30 minutes at RT. For actin staining, Alexa 594 phalloidin was used at a 1:40 dilution for 30 minutes. Cells were rinsed 3 times then mounted with Prolong antifade containing DAPI (Life Technologies). Images of labeled cells were acquired using two microscopy systems: (1) a Zeiss Axioplan II microscope equipped with Plan NEOFLUAR 10x (NA 0.30), F FLUAR 40x (NA 1.30 oil), and Plan APOCHROMAT 63x (NA 1.40 oil) objectives, fitted with a Cooke Sensicam and controlled by the Slidebook software from Intelligent Imaging Innovations (Denver, CO); and (2) a Zeiss LSM 710 laser scanning confocal microscope equipped with a Plan Apo 63x (NA 1.4 oil) objective available at the Scripps Research Institute imaging core facility. Confocal images were analyzed with Imaris (Bitplane). Cell shape parameters were measured with Image J (NIH, https://imagej.nih.gov/ij/). The DAPI (blue) channel in most figures was pseudo-colored as cyan in Adobe Photoshop (Adobe Systems) to make it more visible.

Human myoblast or myotubes were fixed in 2% PFA, then blocked and incubated with anti-RBM3 primary antibody as described above for cell lines. A goat anti-rabbit 488 secondary antibody was added for antibody binding visualization then mounted with VectaShield containing DAPI. Images were captured using a Zeiss inverted fluorescent microscope.

### *In situ* hybridization

Fluorescence *in situ* hybridization was performed as described previously^28^ by using a probe against tRNA-glycine. The oligonucleotide was generated from a template that included a T7 promoter sequence (underlined) – ATAGTGGTGAGCATAGCTGCCTCCTGTCTC – incorporating Digoxigenin (Roche) using the miRVana probe construction kit (Ambion). Cells grown on cover slips were fixed with 4% PFA, permeabilized overnight with 70% ethanol, rehydrated at room temperature for 5 minutes with 2x SSC, then hybridized overnight at 37 °C in RNase free conditions (2x SSC, 30% formamide, 10% dextran, 0.2 mg/ml BSA, 2 mM vanadate ribonucleotide complex, 15 ng of DIG labeled probe). Cells were subsequently washed with 2x SSC/30% formamide 3 × 30 minutes at 37 °C and hybridized probe was detected using an anti-digoxigenin antibody conjugated to FITC (1:200). This step was done in combination with immunostaining, after which cells were mounted with Prolong Gold DAPI (Life Technologies).

### Western blotting

Samples were lysed in a buffer containing 1 mM Tris-HCl (pH 7.4), 1% Triton X-100, 150 mM NaCl, and protease inhibitor cocktail (Roche), then spun to pellet insoluble material. Resulting lysates were assayed for protein concentration, then boiled in NuPAGE SDS Buffer at a final protein centration of 2 mg/ml. Proteins (40ug/lane) were resolved on NuPAGE 4–12% Tris-glycine mini gels using MES running buffer (Life Technologies), then transferred to PVDF membranes (Bio-Rad). After blocking for 30–60 minutes at room temperature, PVDF membranes were incubated overnight at 4 °C with the following antibodies: RhoA (1:200, Santa Cruz), RhoGDI (1:200, Santa Cruz), Rhotekin (1:200, Santa Cruz), β-tubulin (1:200, Santa Cruz), vinculin (1:500, ProSci), CRMP2 (1:500, Abcam), CDC42 (1:500, Upstate), Arp3 (1:500, Upstate), β-actin (1:1000, ProSci), RBM3 (1:2000, made in-house^28^), or vimentin (1:1000, Novus). Following three washes in Tris-buffered saline containing 0.5% Tween 20 (TBST), alkaline phosphatase-conjugated secondary antibodies (goat anti-mouse or rabbit) were added at 1:10,000 for 30 minutes. Immunoblotted membranes were then washed three times in TBST and immunolabeled proteins were detected by chemiluminescence with CDP-star (Roche) and exposed to Kodak autoradiography film. Band intensities were quantified using Image J software and normalized to proteins that did not vary with manipulation of RBM3 expression (eIF6 or β-actin). Uncropped blots are shown in Supplementary Fig. S10 and Supplementary Fig. S11.

### Replating assay

Cells were lifted by trypsinization (Gibco) 48 hours after transfection and replated onto glass coverslips in 24-well dishes. The wells were coated with fibronectin (5–10 µg/ml, Sigma) for 1 hour, then washed in PBS prior to replating. At 0, 60, and 120 minutes post re-plating, cells were either fixed in 4% PFA for immunocytochemistry or harvested in lysis buffer for subsequent Western blot analyses as described above.

### Cell migration real time imaging

B104 cells were transfected with EGFP (con), EGFP combined with RBM3 siRNA (si), or EYFP-RBM3 (o/x) as described above. Cells were replated onto fibronectin-coated (5 μg/ml) glass coverslips, which were then placed in a homeostatic chamber (35–37 °C, 5% CO_2_) mounted onto a Bio-Rad (Zeiss) Radiance 2100 Rainbow laser scanning confocal microscope for live imaging. Images were acquired every 5 minutes. EGFP- and EYFP-transfected cells were tracked, and statistics calculated using Imaris software (Bitplane).

### Wound healing

NIH 3T3 fibroblasts were transfected with siRBM3 duplex (si) or EGFP (con) and plated onto fibronectin-coated coverslips (5 µg/ml). At near confluency, a scratch wound of approximately 300 µm was created with a toothpick and the wound scratch was observed overnight by live imaging as described above. Images were edited in Image J and wound closure was analyzed with Image Pro Plus software (Media Cybernetics).

### Statistics

All statistical analyses in this study were performed using GraphPad Prism 7.03 software for Windows, GraphPad Software, La Jolla California USA, www.graphpad.com. The specific tests performed are listed in the figure legends.

### Data availability

All data generated or analysed during this study are included in this published article (and its Supplementary Information files).

## Electronic supplementary material

Supplementary Information

Supplementary Movie S1

Supplementary Movie S2

Supplementary Movie S3

Supplementary Movie S4

Supplementary Movie S5

Supplementary Movie S6

Supplementary Movie S7

Supplementary Movie S8

Supplementary Movie S9

## References

[1] Derry JM, Kerns JA, Francke U (1995). RBM3, a novel human gene in Xp11.23 with a putative RNA-binding domain. Hum. Mol. Genet..

[2] Danno S (1997). Increased transcript level of RBM3, a member of the glycine-rich RNA-binding protein family, in human cells in response to cold stress. Biochem. Biophys. Res. Commun..

[3] Dresios J (2005). Cold stress-induced protein Rbm3 binds 60S ribosomal subunits, alters microRNA levels, and enhances global protein synthesis. Proc. Natl. Acad. Sci. USA.

[4] Jackson TC (2015). Cold stress protein RBM3 responds to temperature change in an ultra-sensitive manner in young neurons. Neuroscience.

[5] Smart F (2007). Two isoforms of the cold-inducible mRNA-binding protein RBM3 localize to dendrites and promote translation. J. Neurochem..

[6] Sureban, S. M. *et al*. Translation regulatory factor RBM3 is a proto-oncogene that prevents mitotic catastrophe. *Oncogene* (2008). 10.1038/onc.2008.97 PMC2677646 18427544

[7] Cok SJ, Acton SJ, Sexton AE, Morrison AR (2004). Identification of RNA-binding proteins in RAW 264.7 cells that recognize a lipopolysaccharide-responsive element in the 3-untranslated region of the murine cyclooxygenase-2 mRNA. J. Biol. Chem..

[8] Pilotte J, Dupont-Versteegden EE, Vanderklish PW (2011). Widespread regulation of miRNA biogenesis at the Dicer step by the cold-inducible RNA-binding protein, RBM3. PloS one.

[9] Wong, J. J. *et al*. RBM3 regulates temperature sensitive miR-142-5p and miR-143 (thermomiRs), which target immune genes and control fever. *Nucleic Acids Res*, 10.1093/nar/gkw041 (2016). 10.1093/nar/gkw041 PMC4824108 26825461

[10] Fujita J (1999). Cold shock response in mammalian cells. J. Mol. Microbiol. Biotechnol..

[11] Maruyama K, Sato N, Ohta N (1999). Conservation of structure and cold-regulation of RNA-binding proteins in cyanobacteria: probable convergent evolution with eukaryotic glycine-rich RNA-binding proteins. Nucleic Acids Res..

[12] Nishiyama H (1997). Cloning and characterization of human CIRP (cold-inducible RNA-binding protein) cDNA and chromosomal assignment of the gene. Gene.

[13] Nishiyama H (1997). A glycine-rich RNA-binding protein mediating cold-inducible suppression of mammalian cell growth. J. Cell Biol..

[14] Kita H (2002). Modulation of polyglutamine-induced cell death by genes identified by expression profiling. Hum. Mol. Genet..

[15] Wellmann S (2004). Oxygen-regulated expression of the RNA-binding proteins RBM3 and CIRP by a HIF-1-independent mechanism. J. Cell. Sci..

[16] Ryan JC, Morey JS, Ramsdell JS, Van Dolah FM (2005). Acute phase gene expression in mice exposed to the marine neurotoxin domoic acid. Neuroscience.

[17] Wellmann S (2010). The RNA-binding protein RBM3 is required for cell proliferation and protects against serum deprivation-induced cell death. Pediatr. Res..

[18] Zhu X, Zelmer A, Kapfhammer JP, Wellmann S (2016). Cold-inducible RBM3 inhibits PERK phosphorylation through cooperation with NF90 to protect cells from endoplasmic reticulum stress. FASEB J..

[19] Dupont-Versteegden EE (2008). Identification of cold-shock protein RBM3 as a possible regulator of skeletal muscle size through expression profiling. American journal of physiology. Regulatory, integrative and comparative physiology.

[20] Ferry, A. L., Vanderklish, P. W. & Dupont-Versteegden, E. E. Enhanced survival of skeletal muscle myoblasts in response to overexpression of cold shock protein, RBM3. *American journal of physiology. Cell physiolog*y ajpcell.00098.2011 (2011). 10.1152/ajpcell.00098.2011 PMC3154549 21593448

[21] Rosenthal LM (2017). Neuroprotection via RNA-binding protein RBM3 expression is regulated by hypothermia but not by hypoxia in human SK-N-SH neurons. Hypoxia (Auckland, N.Z.).

[22] Chip S (2011). The RNA-binding protein RBM3 is involved in hypothermia induced neuroprotection. Neurobiol. Dis..

[23] Yang HJ (2017). RNA-binding protein RBM3 prevents NO-induced apoptosis in human neuroblastoma cells by modulating p38 signaling and miR-143. Scientific reports.

[24] Zhao W (2014). Spatiotemporal pattern of RNA-binding motif protein 3 expression after spinal cord injury in rats. Cell. Mol. Neurobiol..

[25] Cui Z (2014). Spatiotemporal profile and essential role of RBM3 expression after spinal cord injury in adult rats. J. Mol. Neurosci..

[26] Tong G (2013). Effects of moderate and deep hypothermia on RNA-binding proteins RBM3 and CIRP expressions in murine hippocampal brain slices. Brain Res..

[27] Williams DR (2005). Seasonally hibernating phenotype assessed through transcript screening. Physiol. Genomics.

[28] Pilotte J, Cunningham BA, Edelman GM, Vanderklish PW (2009). Developmentally regulated expression of the cold-inducible RNA-binding motif protein 3 in euthermic rat brain. Brain Res..

[29] Zhou RB, Lu XL, Zhang CY, Yin DC (2017). RNA binding motif protein 3: a potential biomarker in cancer and therapeutic target in neuroprotection. Oncotarget.

[30] Mardakheh FK (2015). Global Analysis of mRNA, Translation, and Protein Localization: Local Translation Is a Key Regulator of Cell Protrusions. Developmental cell.

[31] de Hoog CL, Foster LJ, Mann M (2004). RNA and RNA binding proteins participate in early stages of cell spreading through spreading initiation centers. Cell.

[32] Bergeman J, Caillier A, Houle F, Gagne LM, Huot ME (2016). Localized translation regulates cell adhesion and transendothelial migration. J. Cell Sci..

[33] Andersson MK (2008). The multifunctional FUS, EWS and TAF15 proto-oncoproteins show cell type-specific expression patterns and involvement in cell spreading and stress response. BMC cell biology.

[34] Cervero P, Himmel M, Kruger M, Linder S (2012). Proteomic analysis of podosome fractions from macrophages reveals similarities to spreading initiation centres. Eur. J. Cell Biol..

[35] Mili S, Moissoglu K, Macara IG (2008). Genome-wide screen reveals APC-associated RNAs enriched in cell protrusions. Nature.

[36] Yasuda K (2013). The RNA-binding protein Fus directs translation of localized mRNAs in APC-RNP granules. J. Cell Biol..

[37] Leung KM (2006). Asymmetrical beta-actin mRNA translation in growth cones mediates attractive turning to netrin-1. Nat. Neurosci..

[38] Antar LN, Afroz R, Dictenberg JB, Carroll RC, Bassell GJ (2004). Metabotropic glutamate receptor activation regulates fragile x mental retardation protein and FMR1 mRNA localization differentially in dendrites and at synapses. J Neurosci..

[39] Vanderklish PW (2005). Differential translation and fragile X syndrome. J. Neurophysiol..

[40] Antar LN, Li C, Zhang H, Carroll RC, Bassell GJ (2006). Local functions for FMRP in axon growth cone motility and activity-dependent regulation of filopodia and spine synapses. Mol. Cell. Neurosci..

[41] Hornberg H, Holt C (2013). RNA-binding proteins and translational regulation in axons and growth cones. Frontiers in neuroscience.

[42] De Rubeis S (2013). CYFIP1 coordinates mRNA translation and cytoskeleton remodeling to ensure proper dendritic spine formation. Neuron.

[43] Semple BD, Blomgren K, Gimlin K, Ferriero DM, Noble-Haeusslein LJ (2013). Brain development in rodents and humans: Identifying benchmarks of maturation and vulnerability to injury across species. Prog. Neurobiol..

[44] Peretti D (2015). RBM3 mediates structural plasticity and protective effects of cooling in neurodegeneration. Nature.

[45] Bastide A (2017). RTN3 Is a Novel Cold-Induced Protein and Mediates Neuroprotective Effects of RBM3. Curr. Biol..

[46] Popov VI, Bocharova LS, Bragin AG (1992). Repeated changes of dendritic morphology in the hippocampus of ground squirrels in the course of hibernation. Neuroscience.

[47] von der Ohe CG, Darian-Smith C, Garner CC, Heller HC (2006). Ubiquitous and temperature-dependent neural plasticity in hibernators. J. Neurosci..

[48] Sahai E, Marshall CJ (2003). Differing modes of tumour cell invasion have distinct requirements for Rho/ROCK signalling and extracellular proteolysis. Nature cell biology.

[49] Sanz-Moreno V (2008). Rac activation and inactivation control plasticity of tumor cell movement. Cell.

[50] Friedl P, Wolf K (2010). Plasticity of cell migration: a multiscale tuning model. J. Cell Biol..

[51] Pankova K, Rosel D, Novotny M, Brabek J (2010). The molecular mechanisms of transition between mesenchymal and amoeboid invasiveness in tumor cells. Cell. Mol. Life Sci..

[52] Taddei ML (2014). Mesenchymal to amoeboid transition is associated with stem-like features of melanoma cells. Cell communication and signaling: CCS.

[53] Jones BC (2017). Dual Targeting of Mesenchymal and Amoeboid Motility Hinders Metastatic Behavior. Molecular cancer research: MCR.

[54] Otto A, Collins-Hooper H, Patel A, Dash PR, Patel K (2011). Adult Skeletal Muscle Stem Cell Migration Is Mediated by a Blebbing/Amoeboid Mechanism. Rejuvenation Res.

[55] Collins-Hooper H (2012). Age-related changes in speed and mechanism of adult skeletal muscle stem cell migration. Stem Cells.

[56] Makarenkova, H. P., Gonzalez, K. N., Kiosses, W. B. & Meech, R. Barx2 controls myoblast fusion and promotes MyoD-mediated activation of the smooth muscle alpha actin gene. *J. Biol. Chem*. M807208200 (2009). 10.1074/jbc.M807208200 PMC2685668 19269978

[57] Campion DR (1984). The muscle satellite cell: a review. Int. Rev. Cytol..

[58] Buckingham M (2003). The formation of skeletal muscle: from somite to limb. J. Anat..

[59] Zammit PS (2004). Muscle satellite cells adopt divergent fates: a mechanism for self-renewal?. J. Cell Biol..

[60] Pasut, A., Jones, A. E. & Rudnicki, M. A. Isolation and culture of individual myofibers and their satellite cells from adult skeletal muscle. *Journal of visualized experiments: JoVE*, e50074, 10.3791/50074 (2013). 10.3791/50074 PMC3639710 23542587

[61] Paluch EK, Raz E (2013). The role and regulation of blebs in cell migration. Curr. Opin. Cell Biol..

[62] Bergert M, Chandradoss SD, Desai RA, Paluch E (2012). Cell mechanics control rapid transitions between blebs and lamellipodia during migration. Proc. Natl. Acad. Sci. USA.

[63] Charras, G. & Paluch, E. Blebs lead the way: how to migrate without lamellipodia. *Nature reviews. Molecular cell biology* **9**, 730–736, doi:nrm2453 (2008). 10.1038/nrm2453 18628785

[64] Arthur WT, Burridge K (2001). RhoA inactivation by p190RhoGAP regulates cell spreading and migration by promoting membrane protrusion and polarity. Mol. Biol. Cell.

[65] Gadea G, de Toledo M, Anguille C, Roux P (2007). Loss of p53 promotes RhoA-ROCK-dependent cell migration and invasion in 3D matrices. J. Cell Biol..

[66] Ip JP, Fu AK, Ip NY (2014). CRMP2: functional roles in neural development and therapeutic potential in neurological diseases. Neuroscientist.

[67] Hall C (2001). Collapsin response mediator protein switches RhoA and Rac1 morphology in N1E-115 neuroblastoma cells and is regulated by Rho kinase. J. Biol. Chem..

[68] Arimura N (2005). Phosphorylation by Rho kinase regulates CRMP-2 activity in growth cones. Mol. Cell. Biol..

[69] Mimura F (2006). Myelin-associated glycoprotein inhibits microtubule assembly by a Rho-kinase-dependent mechanism. J. Biol. Chem..

[70] Sun Z (2016). A novel Nogo-66 receptor antagonist peptide promotes neurite regeneration *in vitro*. Mol. Cell. Neurosci..

[71] Verger C, Petrovic M, Imbenotte J (1989). Stimulatory effects of protein synthesis inhibitors on the spreading rate of 3T3 cells. Cell Biol. Int. Rep..

[72] Imbenotte J, Verger C, Sassa S (1985). Modulation of cell attachment to culture support by pH, fibronectin, hemin, and cobalt protoporphyrin. J. Cell. Physiol..

[73] Friedl P, Borgmann S, Brocker EB (2001). Amoeboid leukocyte crawling through extracellular matrix: lessons from the Dictyostelium paradigm of cell movement. J. Leukoc. Biol..

[74] Yamazaki D, Kurisu S, Takenawa T (2009). Involvement of Rac and Rho signaling in cancer cell motility in 3D substrates. Oncogene.

[75] Barranco G (1958). Effect of hypothermia on healing processes of cutaneous wounds. G. Ital. Chir..

[76] Madrid E (2016). Active body surface warming systems for preventing complications caused by inadvertent perioperative hypothermia in adults. The Cochrane database of systematic reviews.

[77] Iaizzo PA, Laske TG, Harlow HJ, McClay CB, Garshelis DL (2012). Wound healing during hibernation by black bears (Ursus americanus) in the wild: elicitation of reduced scar formation. Integrative zoology.

[78] Esclamado RM, Damiano GA, Cummings CW (1990). Effect of local hypothermia on early wound repair. Arch. Otolaryngol. Head Neck Surg..

[79] Harlow HJ, Lohuis T, Anderson-Sprecher C, TDI B (2004). Body surface temperature of hibernating black bears may be related to periodic muscle activity. J. Mammal..

[80] Geiser F (2004). Metabolic rate and body temperature reduction during hibernation and daily torpor. Annu. Rev. Physiol..

[81] Fedorov VB (2011). Modulation of gene expression in heart and liver of hibernating black bears (Ursus americanus). BMC genomics.

[82] Arendt T, Bullmann T (2013). Neuronal plasticity in hibernation and the proposed role of the microtubule-associated protein tau as a “master switch” regulating synaptic gain in neuronal networks. American journal of physiology. Regulatory, integrative and comparative physiology.

[83] Yin J, Yu FS (2008). Rho kinases regulate corneal epithelial wound healing. American journal of physiology. Cell physiology.

[84] Okumura N, Kinoshita S, Koizumi N (2017). The role of Rho kinase inhibitors in corneal endothelial dysnfunction. Curr Pharm Design.

[85] Allen DL, Loh AS (2011). Posttranscriptional mechanisms involving microRNA-27a and b contribute to fast-specific and glucocorticoid-mediated myostatin expression in skeletal muscle. American journal of physiology. Cell physiology.

[86] Yablonka-Reuveni Z, Rivera AJ (1994). Temporal expression of regulatory and structural muscle proteins during myogenesis of satellite cells on isolated adult rat fibers. Dev. Biol..

[87] Relaix, F. & Zammit, P. S. Satellite cells are essential for skeletal muscle regeneration: the cell on the edge returns centre stage. *Development* **139**, 2845–2856, doi:139/16/2845 (2012). 10.1242/dev.069088 22833472

[88] Morata-Tarifa C (2016). Low adherent cancer cell subpopulations are enriched in tumorigenic and metastatic epithelial-to-mesenchymal transition-induced cancer stem-like cells. Scientific reports.

[89] Jonsson L (2011). Low RBM3 protein expression correlates with tumour progression and poor prognosis in malignant melanoma: an analysis of 215 cases from the Malmo Diet and Cancer Study. J Transl Med.

[90] Jonsson L (2011). High RBM3 expression in prostate cancer independently predicts a reduced risk of biochemical recurrence and disease progression. Diagnostic pathology.

[91] Ye F (2017). High RNA-binding motif protein 3 (RBM3) expression is independently associated with prolonged overall survival in intestinal-type gastric cancer. Med Sci Monit..

[92] Siesing, C. *et al*. High RBM3 expression is associated with an improved survival and oxaliplatin response in patients with metastatic colorectal cancer. *PLoS One*. **12**, 10.1371/journal.pone.0182512 (2017). 10.1371/journal.pone.0182512 PMC5553773 28800641

[93] Jang HH, Lee HN, Kim SY, Hong S, Lee WS (2017). Expression of RNA-binding Motif Protein 3 (RBM3) and Cold-inducible RNA-binding protein (CIRP) Is Associated with Improved Clinical Outcome in Patients with Colon Cancer. Anticancer Res..

[94] Boman K (2013). Decreased expression of RNA-binding motif protein 3 correlates with tumour progression and poor prognosis in urothelial bladder cancer. BMC urology.

[95] Jogi A (2009). Nuclear expression of the RNA-binding protein RBM3 is associated with an improved clinical outcome in breast cancer. Mod. Pathol..

[96] Hjelm B (2011). High nuclear RBM3 expression is associated with an improved prognosis in colorectal cancer. Proteomics. Clinical applications.

[97] Wolf K (2003). Compensation mechanism in tumor cell migration: mesenchymal-amoeboid transition after blocking of pericellular proteolysis. J. Cell Biol..

[98] Torka R, Thuma F, Herzog V, Kirfel G (2006). ROCK signaling mediates the adoption of different modes of migration and invasion in human mammary epithelial tumor cells. Exp. Cell Res..

[99] Kosla J (2013). Metastasis of aggressive amoeboid sarcoma cells is dependent on Rho/ROCK/MLC signaling. Cell communication and signaling: CCS.

[100] Sadok A (2015). Rho kinase inhibitors block melanoma cell migration and inhibit metastasis. Cancer Res..

[101] Zhang HT (2013). Differential expression of the RNA-binding motif protein 3 in human astrocytoma. Chin. Med. J. (Engl)..

[102] Grupp K (2014). High RNA-binding motif protein 3 expression is an independent prognostic marker in operated prostate cancer and tightly linked to ERG activation and PTEN deletions. Eur. J. Cancer.

[103] Ehlen A (2011). RBM3-regulated genes promote DNA integrity and affect clinical outcome in epithelial ovarian cancer. Translational oncology.

[104] Fan G (2015). A quantitative proteomics-based signature of platinum sensitivity in ovarian cancer cell lines. Biochem. J..

[105] Venugopal A (2016). RNA binding protein RBM3 increases beta-catenin signaling to increase stem cell characteristics in colorectal cancer cells. Mol. Carcinog..

[106] Thornton JE, Gregory RI (2012). How does Lin28 let-7 control development and disease?. Trends Cell Biol..

[107] Zhou J, Ng SB, Chng WJ (2013). LIN28/LIN28B: an emerging oncogenic driver in cancer stem cells. Int. J. Biochem. Cell Biol..

[108] Liu K (2015). Let-7a inhibits growth and migration of breast cancer cells by targeting HMGA1. Int. J. Oncol..

[109] Spolverini, A., Fuchs, G., Bublik, D. R. & Oren, M. let-7b and let-7c microRNAs promote histone H2B ubiquitylation and inhibit cell migration by targeting multiple components of the H2B deubiquitylation machinery. *Oncogene*10.1038/onc.2017.187 (2017). 10.1038/onc.2017.187 PMC5600258 28604753

[110] Rando TA, Blau HM (1997). Methods for myoblast transplantation. Methods Cell Biol..

[111] Moyle LA, Zammit PS (2014). Isolation, culture and immunostaining of skeletal muscle fibres to study myogenic progression in satellite cells. Methods Mol. Biol..

