# Supplementary material for: Morphoregulatory functions of the RNA-binding motif protein 3 in cell spreading, polarity and migration
Source: Sci Rep. 2018 May 9;8:7367. doi: 10.1038/s41598-018-25668-2 (PMC5943363; doi:10.1038/s41598-018-25668-2)
Supplement: Supplementary file 1 — Supplementary Information [file 41598_2018_25668_MOESM1_ESM.pdf]

Supplementary information

Morphoregulatory functions of the RNA-binding motif protein 3 in cell spreading, polarity and migration

Pilotte J., Kiosses W., Chan S.W., Makarenkova H.P., Dupont-Versteegden E., Vanderklish P.W.

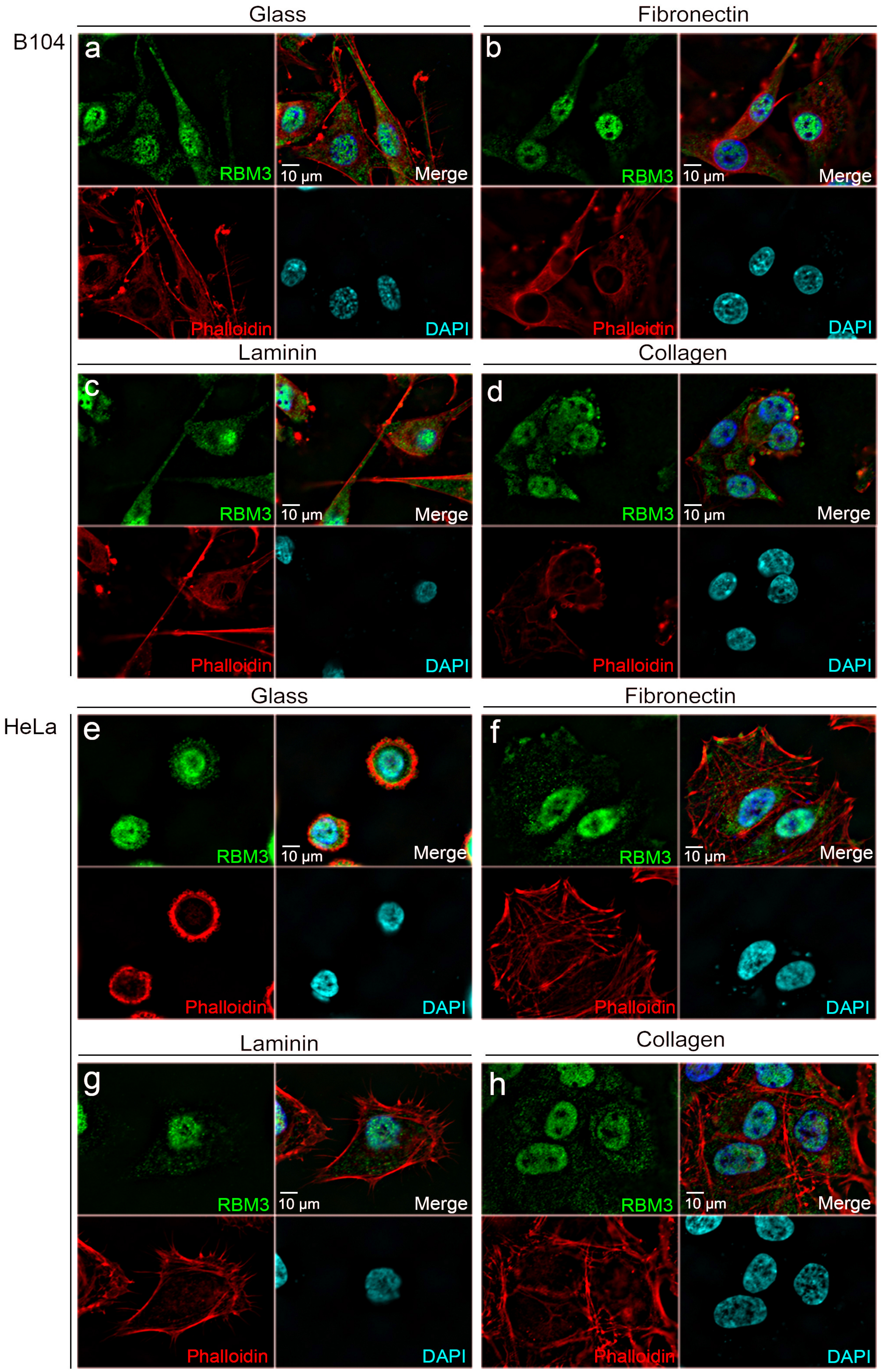

Figure S1. RBM3 adopts a nucleocytoplasmic expression pattern in multiple cell types and on multiple substrata at later time points in culture. B104 (**A-D**) and HeLa (**E-H**) cells were plated onto glass, fibronectin, laminin, or collagen and fixed for immunolabeling of RBM3 (green) and counterstaining for nuclei (DAPI, blue) and F-actin (phalloidin, red) at 3 hrs post plating. Cell types and plating substrates are indicated. In all conditions, a nucleocytoplasmic distribution of RBM3 is evident at 3 hrs post plating.

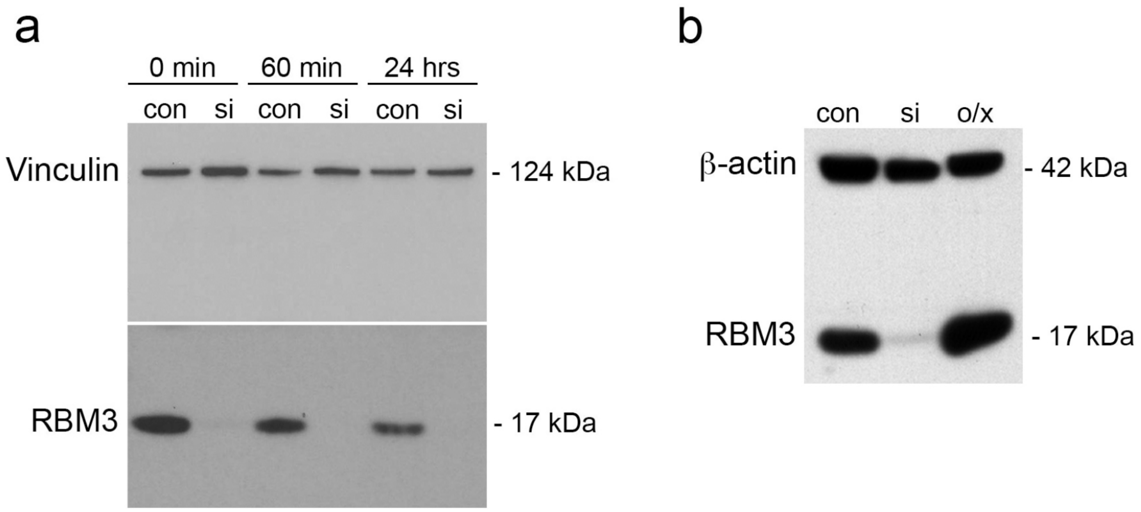

Figure S2. Manipulation of RBM3 expression. (**A**) Western blots showing levels of RBM3 in B104 cells at several time points during a replating assay. Virtually complete knockdown of RBM3 is maintained for at least 24 hrs after replating of siRNA treated cells. (**B**) Representative Western blots showing RBM3 expression levels in B104 cells maintained under control (con), RBM3 knockdown (si) and overexpression (o/x) conditions.

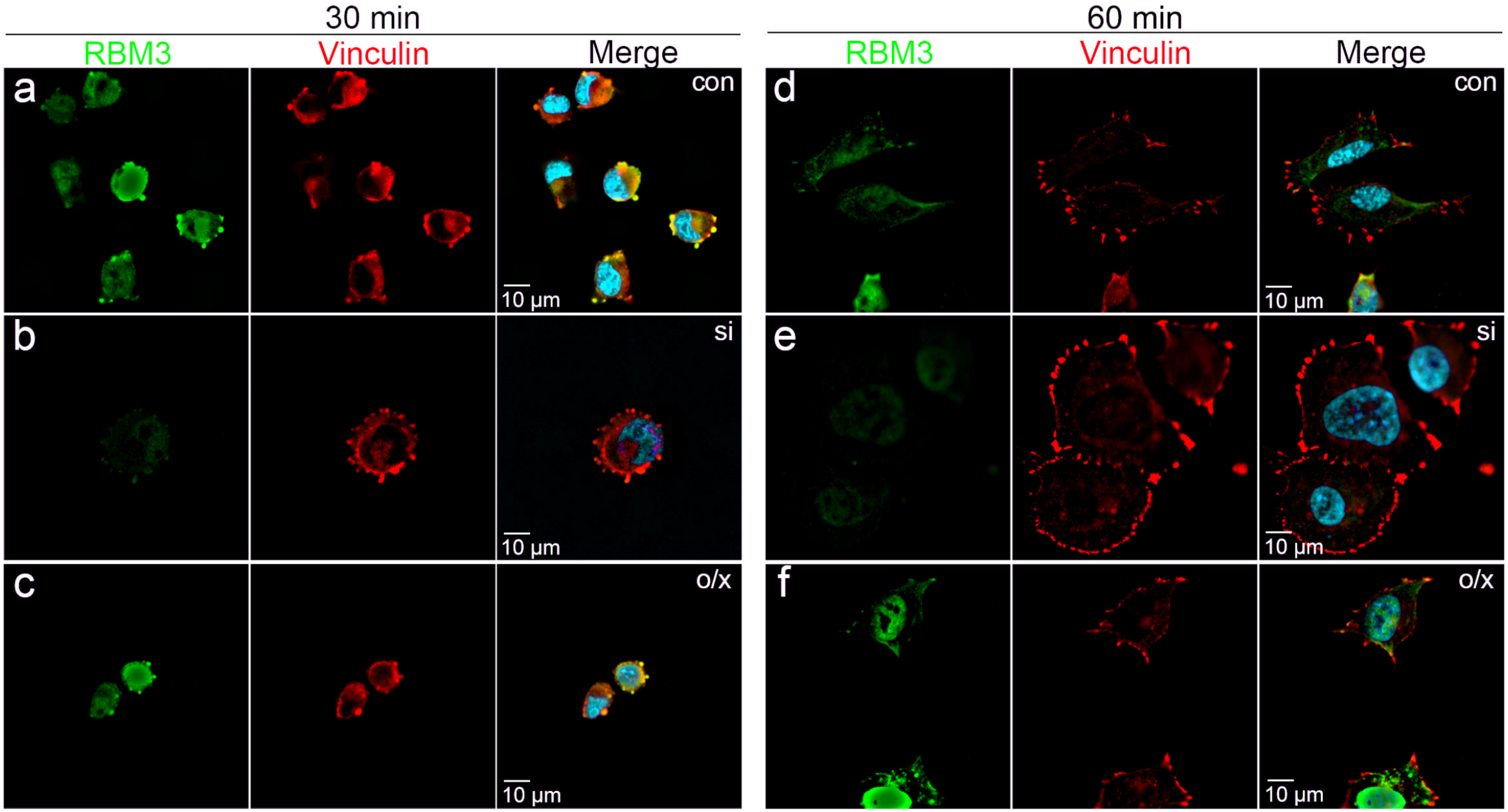

Figure S3. Perturbation of RBM3 expression alters cell morphology. B104 cells were transfected with empty vector (control; **A,D**), an siRNA to RBM3 (**B,E**), or a CMV-based expression vector for EYFP-RBM3 (**C,F**), then replated 48 hrs later onto fibronectin-coated glass cover slips and fixed at 30 min (**A-C**) and 60 min (**D-F**) post plating. Each set of 3 images shows immunolabeling for RBM3 (green), vinculin (red), and an overlay of these channels with the nuclear stain DAPI (blue). In cells fixed at 30 min post plating, vinculin-positive foci resembling SICs were still present in all three conditions; however, RBM3 knockdown cells were already becoming more spread. In cells fixed at 60 min, control and RBM3 overexpressing cells exhibited a polar cell morphology with growth cone-like protrusions containing RBM3 and vinculin, whereas cells lacking RBM3 exhibited a rounded, highly spread morphology with a discontinuous ring of vinculin-positive foci that resembled focal adhesions.

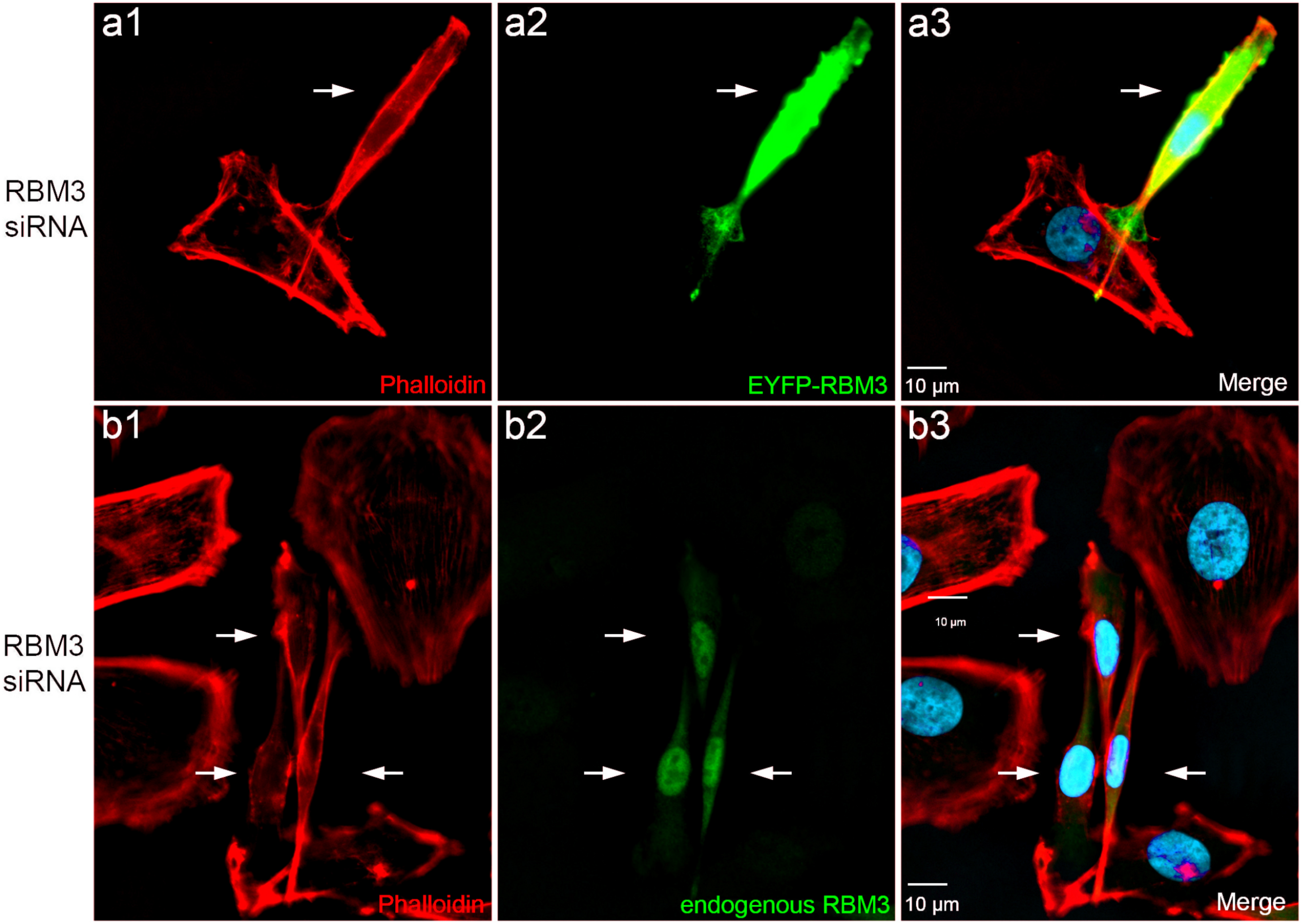

Figure S4. Rescue of RBM3 siRNA-induced changes in B104 cell morphology. B104 cells were transfected with RBM3 siRNA plus or minus an EYFP-RBM3 expression construct for 48 hrs, then replated onto fibronectin-coated glass coverslips and fixed at 3 hrs post plating. (**A1-3**) Images of F-actin (phalloidin, red) and EYFP-RBM3 (green) in neighboring B104 cells in which one cell expressed EYFP-RBM3 (arrow) and the other did not. Expression of exogenous EYFP- RBM3 converted the highly spread polygonal cell morphology into a bipolar shape. (**B1-3**) Images of F-actin and endogenous RBM3 (green) in neighboring B104 cells. Cells in which RBM3 knockdown was inefficient (arrows) adopted an elongated, bipolar morphology, whereas those lacking detectable endogenous RBM3 where highly spread and rounded or polygonal.

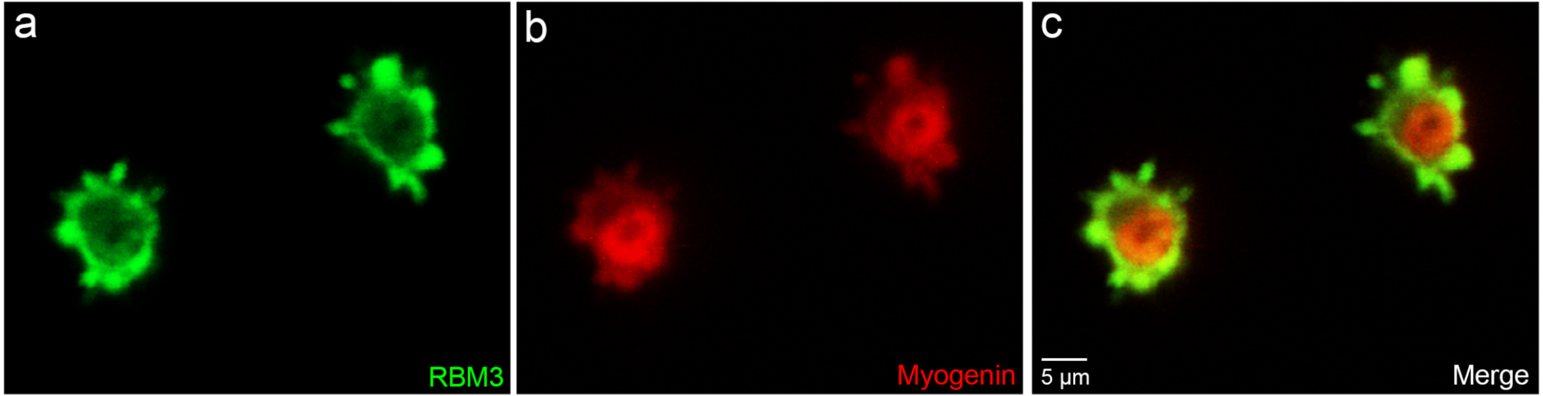

Figure S5. RBM3 is expressed in primary myoblasts. (**A-C**) RBM3 expression in myoblasts co-expressing myogenin, a marker of the activated state. Primary myoblasts were seeded onto collagen coated chamber slides. Cells were cultured in growth medium (DMEM/F10, containing FGF2 and 20%FBS) for three days. To induce cell differentiation, growth medium was replaced with DMEM containing 5% horse serum. Images show expression of RBM3 (**A**, green), myogenin (**B**, red), and the overlay of these channels (**C**, yellow).

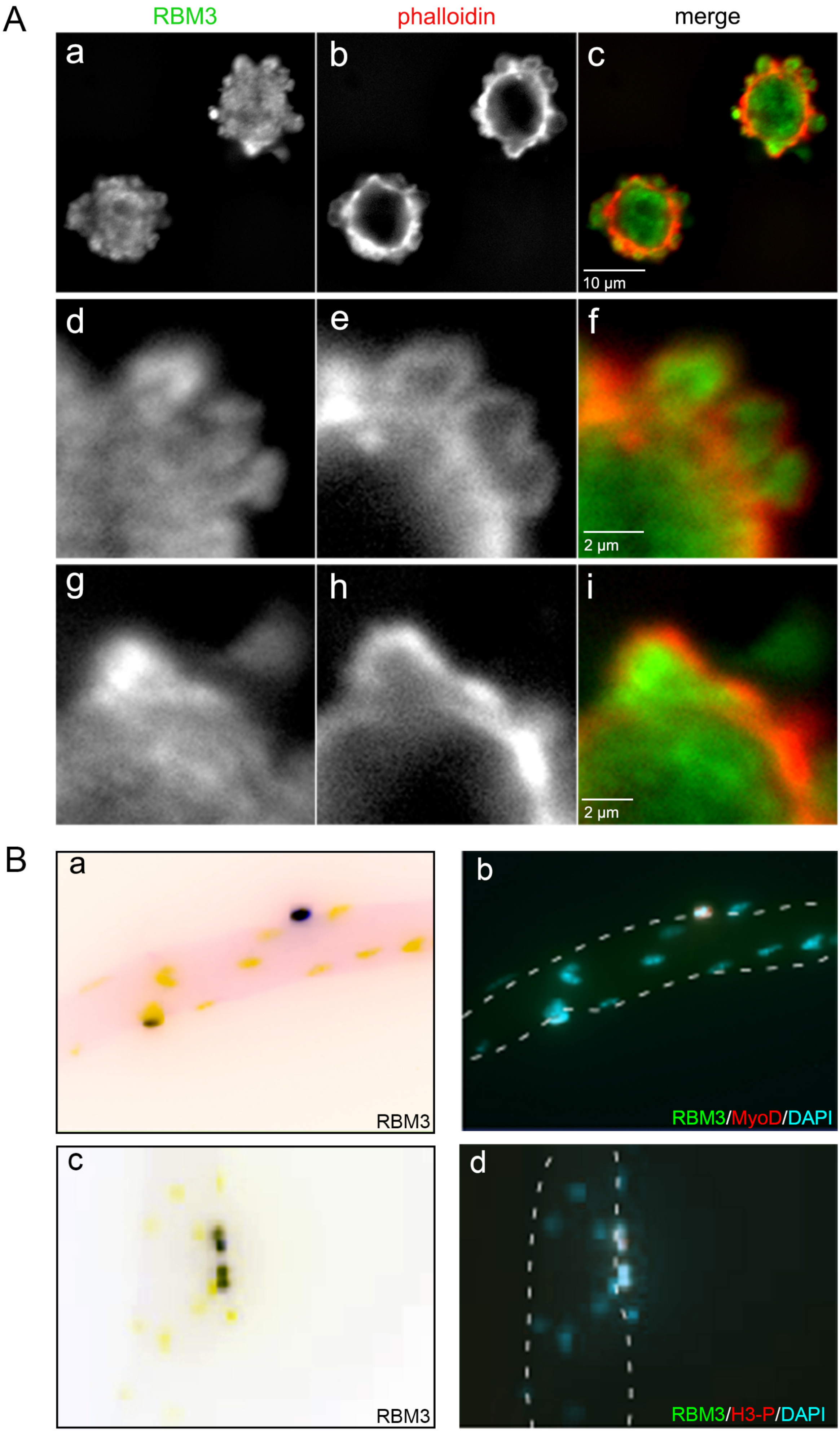

Figure S6. Dynamic localization of RBM3 in differentiating primary myoblasts. (**A**) Black and white images from Figure 5A, B and D showing RBM3 (**a, d, g)**, F-actin (phalloidin; **b, e, h**) and overlay images for RBM3 and phalloidin (merge; **c, f, i)**. (**B**) Inverted images from Figure 5G, H showing the outline of the myotube explant at 24 hrs in culture in which activated satellite cells migrating along the myotube border (dashed outline) co-express MyoD (red; **a**) and RBM3 (green; **b**), or histone H3 phosphate (H3-P, red; **c**) and RBM3 (green; **d**).

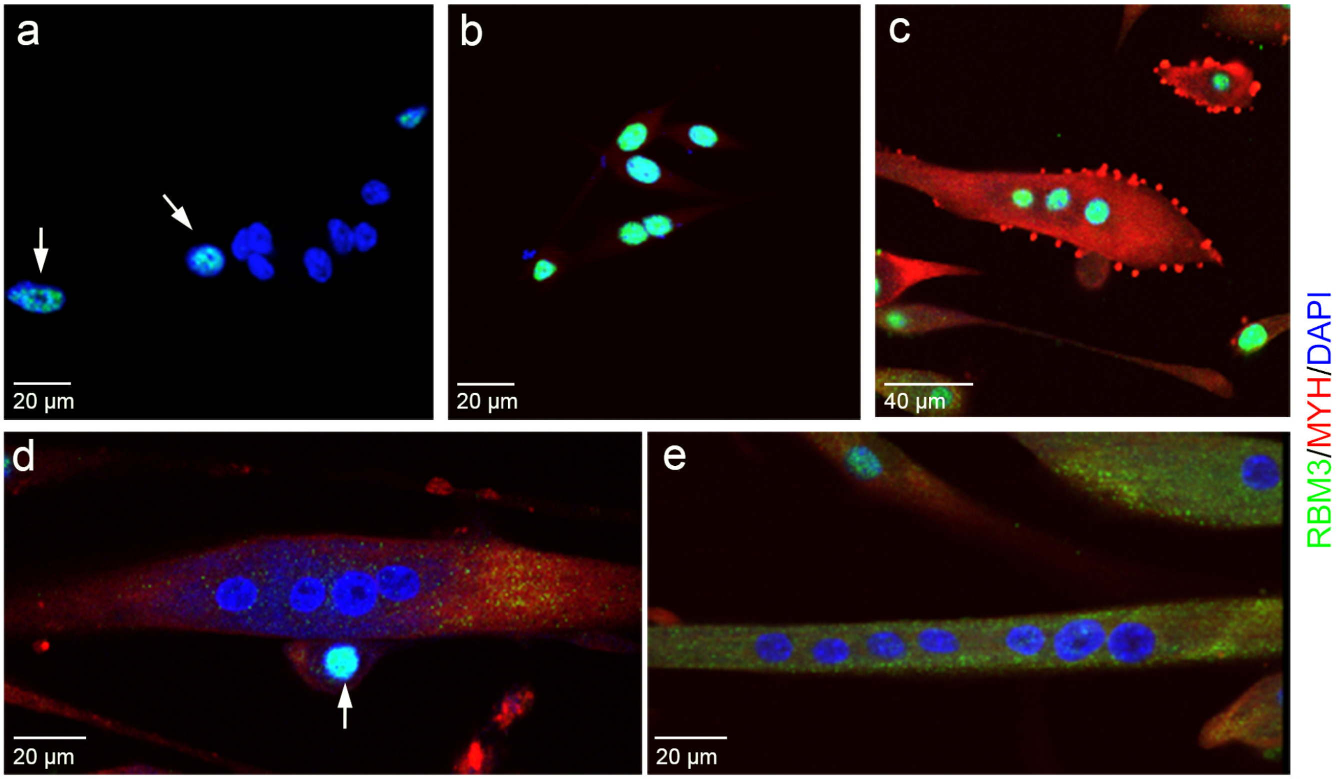

Figure S7. RBM3 is expressed in myoblasts cells during migration and fusion, and in myotubes formed from these cells. (**A**) Image of myoblast cells cultured in non-differentiating conditions then labeled with DAPI (blue) and immunostained for RBM3 (green). Some cells (arrows) spontaneously express RBM3. (**B**) Image of myoblast cells induced to differentiate by serum withdrawal; after 3 hrs, all cells express RBM3 (green). (**C**) Image of cells cultured as in panel B at 24 hrs post serum withdrawal. Newly formed myotubes are seen expressing myosin heavy chain (MYH, red), and peripheral blebbing can be seen. RBM3 is expressed in nuclei at this late stage in culture. (**D**) Images myotubes at 48 hrs post serum withdrawal showing expression of RBM3 (green) and myosin heavy chain (red) in the cytoplasm. An activated satellite cell can be seen fusing to the myotube and expressing RBM3 (arrow). (**E**) Image of elongated myotube exhibiting cytoplasmic RBM3 expression.

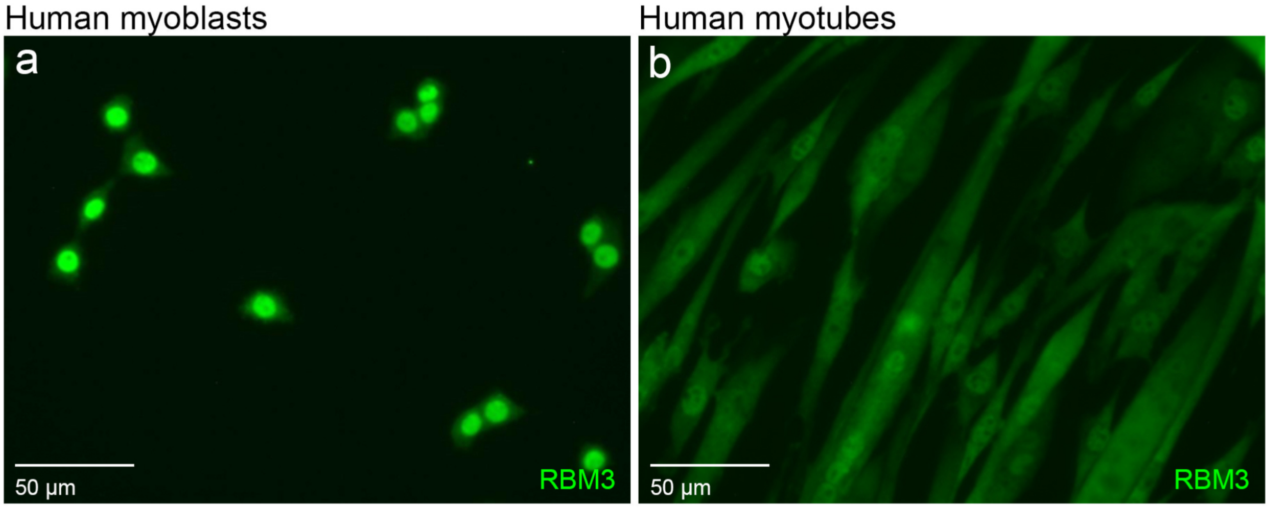

Figure S8. RBM3 is expressed in human myoblasts and myotubes. Images showing RBM3 expression (green) in primary human myoblasts (**A**) and in myotubes formed from human myoblasts (**B**).

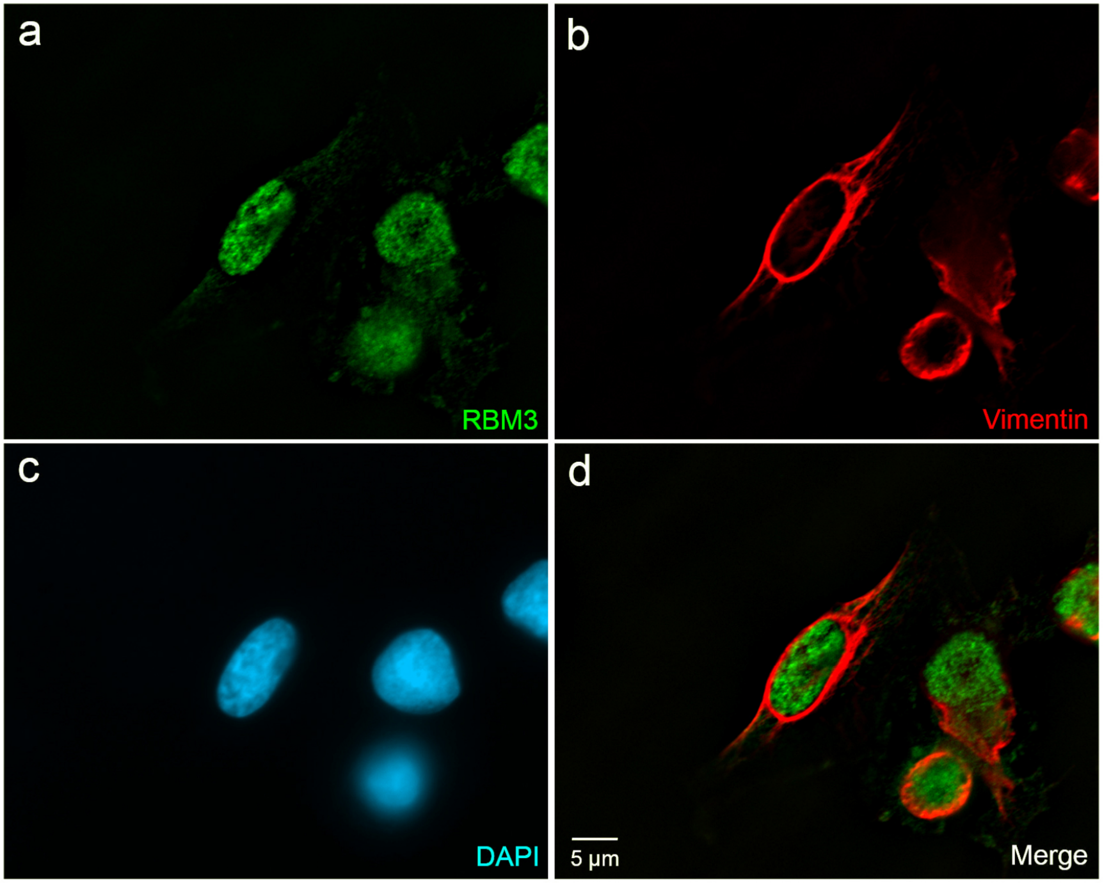

Figure S9. B104 neuroblastoma cells express vimentin. (***A-C***) Images of B104 cells immunofluorescently labeled for RBM3 (green) and vimentin (red), and counterstained with DAPI (blue). (**D**) Overlay of RBM3 and vimentin (Merge).

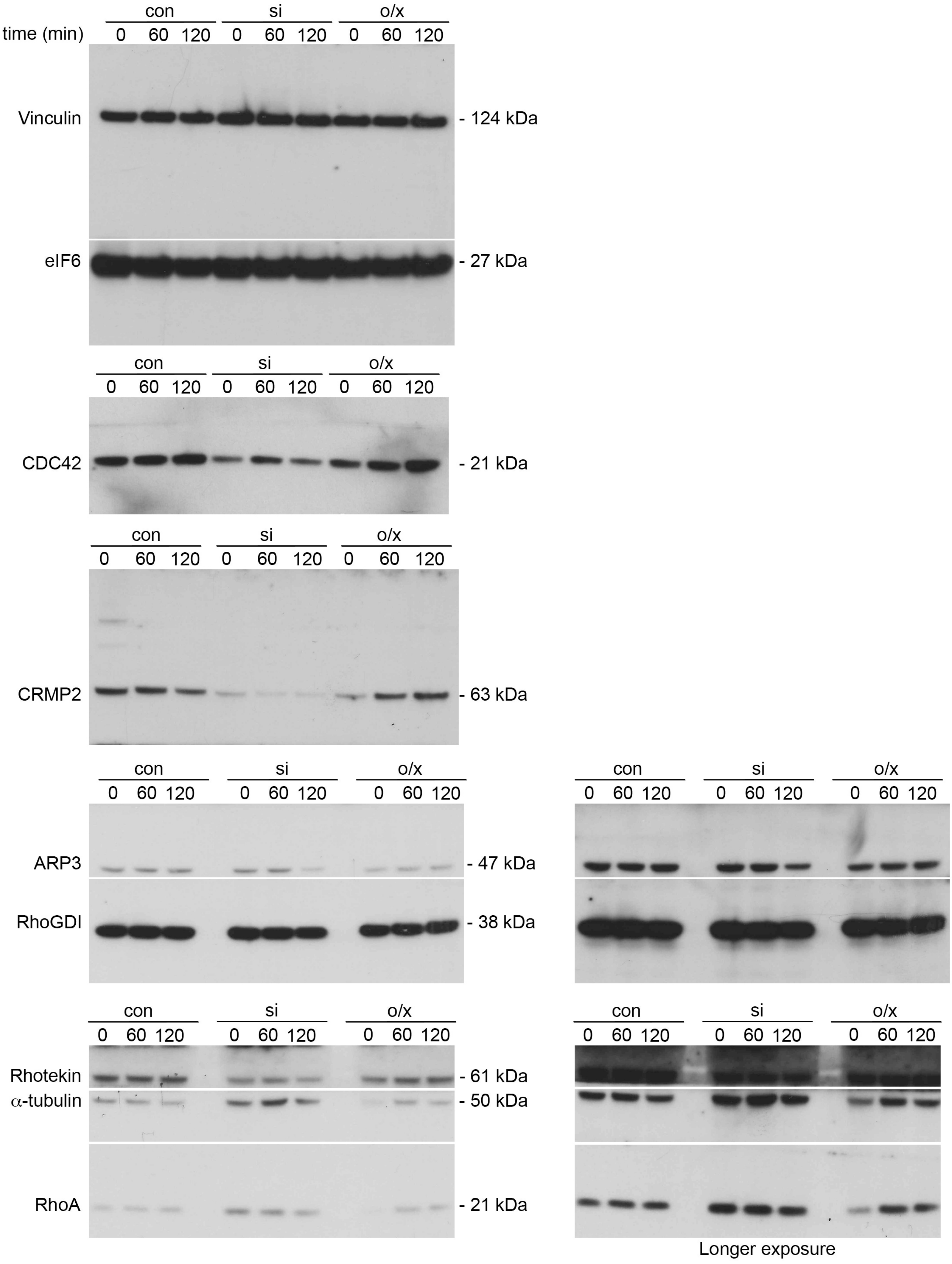

Figure S10. Full length western blots from Figure 7A. Western blots showing levels of Vinculin, EIF6, CDC42, CRMP2, ARP3, RhoGDI, Rhotekin, α-tubulin and RhoA in B104 cells at several time points during a replating assay under control (con), RBM3 knockdown (si) and overexpression (o/x) conditions. Darker and lighter exposure blots used for quantification are shown.

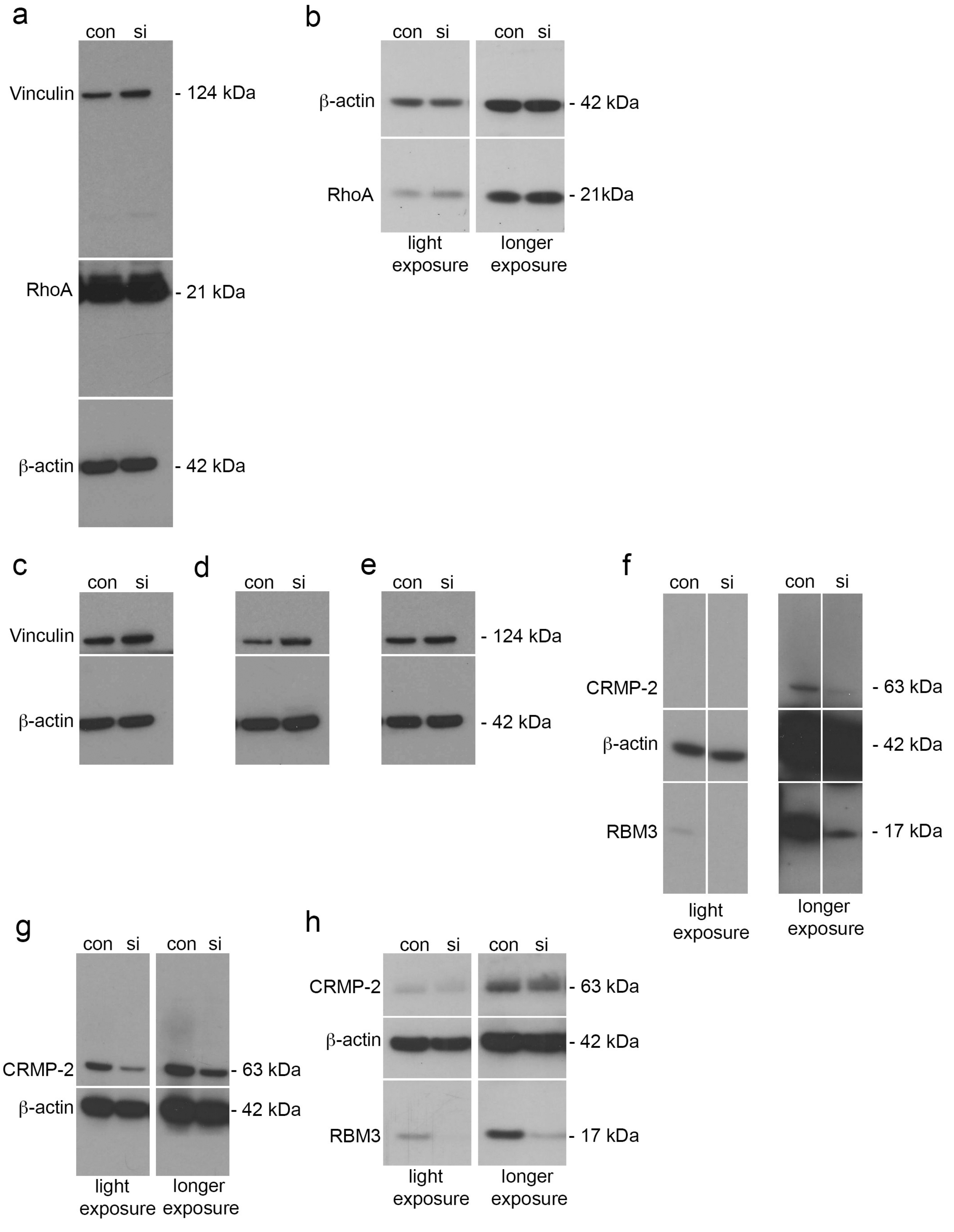

Figure S11. Supplemental western blots from Figure 7B quantification. (**A**) Western blots showing levels of Vinculin and RhoA, and normalized to β-actin for control (con) and RBM3 knockdown (si) in B104 neuroblastoma cells. (**B**) Western blots showing levels of RhoA and normalized to β-actin. (**C-E**) Western blots showing levels of Vinculin and normalized to β-actin. (**F-H**) Western blots showing levels of CRMP2 and RBM3 normalized to β-actin. Lighter and darker exposure blots used for quantification are shown.

Supplementary Movie Legends

Supplementary Movie S1 Live imaging of control B104 cells transfected with EGFP. The tracked migration path is superimposed.

Supplementary Movie S2 Live imaging of a separate set of EGFP-transfected control B104 cells.

Supplementary Movie S3 Live imaging of RBM3 knockdown B104 cells transfected with RBM3 siRNA and EGFP. The tracked migration paths are superimposed.

Supplementary Movie S4 Live imaging of RBM3 knockdown B104 cells shown in Movie S3, but without superimposed pathways.

Supplementary Movie S5 Live imaging of a separate pair of RBM3 knockdown B104 cells.

Supplementary Movie S6 Live imaging of RBM3 overexpressing B104 cells transfected with EYFP-RBM3. The tracked migration paths are superimposed.

Supplementary Movie S7 Live imaging of a separate set of RBM3 overexpressing B104 cells transfected with EYFP-RBM3.

Supplementary Movie S8 Live imaging of wound healing in control 3T3 fibroblasts.

Supplementary Movie S9 Live imaging of wound healing in RBM3 knockdown 3T3 fibroblasts.
